# Supplementary material for: When good for business is not good enough: Effects of pro-diversity beliefs and instrumentality of diversity on intergroup attitudes
Source: PLoS One. 2020 Jun 1;15(6):e0234179. doi: 10.1371/journal.pone.0234179 (PMC7263624; doi:10.1371/journal.pone.0234179)
Supplement: S1 File — (PDF) [file pone.0234179.s001.pdf]

S1 File. Pretest results.

The pretest was situated in the context of intergroup relations between different university student groups at a German university. We focused on educational background diversity, i.e., diversity with regard to differences in individuals' chosen field of study/degree program. Participants were psychology students who were told that they would be collaborating with economics students. Economics students are a highly relevant outgroup for psychology students in Germany, because both groups compete for jobs in some areas. Moreover, economics students tend to be consensually devalued among other students in Germany; they are often seen as high in competence but low in warmth (Honert, 2006).

In order to analyze the interaction between pro-diversity beliefs and instrumentality of diversity on intergroup attitudes, we first measured participants' pro-diversity beliefs. We then manipulated instrumentality by experimentally varying the outcome of a simulated cooperative task within educationally diverse dyads (psychology students vs. economics students). Finally, intergroup attitudes were measured with items tapping prejudice and positive feelings towards the outgroup, economics students.

Method

Participants were randomly assigned to one of three different conditions: detrimental, instrumental, and control condition. All participants first answered items measuring their pro-diversity beliefs. After that, they were asked to work on four brainstorming tasks related to an advertisement. In the detrimental condition as well as in the instrumental condition, participants were told that they would cooperate with a different student of economics in each of the four tasks. We manipulated the instrumentality of these interactions, that is, feedback about the joint performance was varied across the conditions. Feedback in the detrimental condition was designed to indicate that the interaction was non-useful, that is, that it led to results that were below average. Feedback in the instrumental condition was designed to indicate that the interaction was useful, that is, that it led to results that were above average. In the control condition participants worked alone and received individual neutral feedback. After having completed the brainstorming tasks, attitudes toward students of economics were measured with two scales (i.e., feeling thermometer and prejudice ratings).

In total 80 participants participated in the experiment in return for course credit. Two participants had to be excluded from analyses because they were not students of psychology.

Of the remaining 78 participants 56 were women (mean age = 22.8, *SD* = 3.8). Participants were equally distributed across the three conditions. The small sample size is due to students' preference for online studies and the resulting difficulties in recruiting students for on-site lab-experiments during that time.

The study was announced as a study on product advertising and took part in the social psychology lab of the department of psychology at a medium-sized German university. Groups of three to four psychology students participated simultaneously in one experimental session. Upon arrival in the lab, the experimenter told participants that they would be working individually (control condition) or in cooperation with one of four different students of economics (detrimental and instrumental conditions) on four dyadic brainstorming tasks related to product advertising. Moreover, they were told that the focus of the study lay on how the unique views of psychology and economics students jointly contribute to performance in product advertising. After that, the experimenter explained that the economics students would be working on the task from another university building, and that the cooperation would be computer-mediated; participants were told that their answers would be combined with the answers of an economics student. To increase plausibility of the computer-mediated cooperation participants saw a photo of their interaction partner and had their photo taken also that would be ostensibly shown to their collaboration partner. Moreover, we integrated delays of varying lengths into the sequence of the brainstorming task to make participants believe that they had to wait for their interaction partners' answers. In the control condition participants were told that they would be working on the tasks on their own. Next, participants filled in a computer administered questionnaire that included questions on demographics, a number of distractor items related to advertising, as well as items measuring pro-diversity beliefs, before proceeding to the brainstorming task.

In the task participants ran through four trials. In each trial they were asked to come up with ten words that could be used to promote four different innovative products (a heated office chair, caffeinated tooth paste, sneakers with an integrated GPS module, mint water) in a print advertisement. In the detrimental and the instrumental conditions, participants were told that the economics student they were paired with had to complete the same task. Since the participants were made to believe that the researchers' interests lay on the joint performance of psychology and economics students, participants were asked to bring their unique perspective as psychology students to the task, and were told that the same would be asked of the economics student. A joint group score would then be calculated by adding up the number

of unique words generated in each trial (i.e., words that were only named once; Homan, van Knippenberg, Van Kleef, & De Dreu, 2007).

During the computer-mediated task participants saw a description of the products along with instructions. In both experimental conditions, participants additionally saw a photo of a different supposed interaction partner in each trial (photos displayed four actors in a similar age range as participants, two of them were female and two male). After submitting their responses, participants received feedback about their individual (control condition) or their group performance (detrimental and instrumental conditions). In the control condition participants were given minimal feedback, merely saying "You have listed 10 words." for all trials. In the instrumental condition participants read "You and your interaction partner together listed 18 (19/18/20 in trials 2 to 4) unique words. 2 (1/2/0 in trials 2 to 4) words were similar to each other and will not count to your performance score." In the detrimental condition participants read "You and your interaction partner together listed 10 (11/10/12 in trials 2 to 4) unique words. 10 (9/10/8 in trials 2 to 4) words were similar to each other and will therefore not count to your performance score." In order to manipulate instrumentality, participants in the detrimental condition and instrumental condition additionally received the following information classifying their group's result: "Previous results indicate that performance scores below 12 unique words can be considered as below average, 13 to 17 unique words as average, and above 18 unique words as above average.".

After completion of the brainstorming task, participants were asked to fill in a questionnaire. In the detrimental and the instrumental condition participants first evaluated the collaboration with economics students in terms of its instrumentality (the answers functioned as a manipulation check). All participants were then asked to answer a number of feeling thermometers and prejudice items for economics students, as well as for a number of different student groups (i.e., art, philosophy, and pedagogics students). We included items for these additional groups to minimize demand characteristics. After having completed the questionnaire participants were thanked and debriefed.

Unless otherwise indicated, all items were answered on 5-point-scales ranging from 1 = *do not agree* at all to 5 = *totally agree*. Items measuring *pro-diversity beliefs* were nested within the context of universities. They were measured with seven items (e.g., 'Everyone would profit from more multidisciplinarity within the university.'; α = .639). The manipulation check *perceived instrumentality* was measured with two items (e.g., 'How useful would you rate the collaboration with your partner?'; $r = .789$, $p < .001$; scaling from 1

= *not useful at all* to 5 = *very useful*). Attitudes towards economics students were measured with *one feeling thermometer item* (Haddock, Zanna, & Esses, 1993): ‘In general, how would you rate your feelings towards economics students?’ (scaling from 0 = *very cold* to 10 = *very warm*) as well as nine items measuring *prejudice towards economics student* (e.g., ‘I would not like to move in with an economics student.’; α = .755).

As mentioned above, we also included feeling thermometer and prejudice measures targeting four additional student outgroups (i.e., art, philosophy, law, and pedagogics). In addition, we measured intergroup contact with different student groups, political orientation, and ethnic pro-diversity beliefs nested in a societal context (e.g., ‘It is better for a country if there is a variety of cultures.’). These variables were not considered for our main analyses.

Results and Discussion

We first analyzed whether our manipulation was successful. This was the case, as participants in the detrimental condition perceived less instrumentality (M = 2.63, SD = 0.81) than participants in the instrumental condition (M = 3.98, SD = 1.07; $t(50)$ = -5.12, p < .001; d = 1.43).

To test our hypothesis, we analyzed whether the effect of pro-diversity beliefs on attitudes towards students of economics (i.e. prejudice and feeling thermometer) was moderated by instrumentality (dummy coded with two variables: a) control as a baseline condition vs. detrimental and b) control vs. instrumental). We expected that pro-diversity beliefs would positively relate to favorable attitudes towards students of economics when diversity was perceived as instrumental but not as detrimental. To test our assumptions, we used Model 1 in the process macro for SPSS (Hayes, 2013) with dummy coding for the moderator. As predicted, results indicated that the effect of pro-diversity beliefs on prejudice was moderated by instrumentality. The interaction terms for pro-diversity beliefs and the dummy-coded experimental conditions significantly added to the predictive value of the regression model (ΔR^2 = .119, $F(2, 72)$ = 4.97, p = .010). Analyses of conditional effects revealed that pro-diversity beliefs were associated with less prejudice in the control condition (b = -0.431, SE = 0.193, p = .028, $CI_{95\%}$ = -0.815, -0.047), with more prejudice in detrimental condition (b = 0.562, SE = 0.252, p = .029, $CI_{95\%}$ = 0.060, 1.065), and, surprisingly, were unrelated with prejudice in the instrumental condition (b = 0.055, SE = 0.323, p = .866, $CI_{95\%}$ = -.588, .698).

A similar pattern was found when predicting scores on the feeling thermometer: Again the effect of pro-diversity beliefs was moderated by instrumentality ($\Delta R^2 = .110$, $F(2, 72) = 4.69$, $p = .012$). Analyses of conditional effects revealed that pro-diversity beliefs exerted no effects on favorable attitudes in the control condition ($b = 0.921$, $SE = 0.711$, $p = .200$, $CI_{95\%} = -0.497, 2.339$) nor in the instrumental condition ($b = -0.035$, $SE = 1.192$, $p = .977$, $CI_{95\%} = -2.410, 2.341$), but were associated with less favorable attitudes in the detrimental condition ($b = -2.652$, $SE = 0.931$, $p = .006$, $CI_{95\%} = -4.507, -0.796$).

Taken together, our results indicate that detrimental collaboration with outgroup members (economics students) on a team task deteriorated attitudes towards this group for individuals holding strong beliefs in the instrumentality of educational diversity at the university. Detrimental diversity thus led to a deterioration of attitudes towards economics students among psychology students holding pro-diversity beliefs. In other words, we found support for the hypothesis that the effect of pro-diversity beliefs on intergroup attitudes is moderated by the perceived instrumentality of diversity. Interestingly, we did not observe a prejudice-reducing effect of instrumental collaboration in diverse teams in general nor an effect of pro-diversity beliefs after instrumental collaboration. This, however, may be partially due to the specific intergroup context considered in this study, since negative attitudes towards economics students are relatively commonplace and even perceived as socially desirable among psychology students.

Supplemental analyses

Although we primarily included the items measuring attitudes towards various other student outgroups as distractors, we additionally tested whether the effect of pro-diversity beliefs on attitudes towards the other student outgroups was moderated by instrumentality of collaboration with students of economics. We did not find any significant interaction effects of pro-diversity beliefs and instrumentality for prejudice and feeling thermometer scores towards art students (*p's* > .317). We did, however, find a significant interaction effect on prejudice towards philosophy students ($\Delta R^2 = .073$, $F(2, 72) = 3.19$, $p = .047$). Analyses of conditional effects revealed that there was no significant effect of pro-diversity beliefs on prejudice towards philosophy students in the control condition ($b = -0.024$, $SE = 0.183$, $p = .891$, $CI_{95\%} = -0.339, 0.389$), a marginally significant negative effect in the detrimental condition ($b = -0.429$, $SE = 0.239$, $p = .077$, $CI_{95\%} = -0.078, 0.639$), and a significant negative effect in the instrumental condition ($b = -0.828$, $SE = 0.306$, $p = .009$, $CI_{95\%} = -1.438, -0.218$).

For feeling thermometer scores toward philosophy students the interaction between pro-diversity beliefs and instrumentality was marginally significant ($\Delta R^2 = 0.077$, $F(2, 72) = 3.10$, $p = .051$). Probing for conditional effects, we only found a significant positive effect of pro-diversity beliefs on feelings thermometer scores for philosophy students in the detrimental condition ($b = -2.551$, $SE = 1.017$, $p = .014$, $CI_{95\%} = 0.524, 4.579$) but no significant effects in the control ($b = -0.112$, $SE = 0.777$, $p = .886$, $CI_{95\%} = -1.662, 1.438$) or the instrumental condition ($b = -1.105$, $SE = 1.302$, $p = .399$, $CI_{95\%} = -3.700, 1.490$). Note, however, that the results for philosophy students differ from the pattern of results found for students of economics. Pro-diversity beliefs thus decreased prejudice towards philosophy students when students of economics were depicted as instrumental and increased favorable attitudes towards philosophy students when students of economics were depicted as non-instrumental. The pattern of results for attitudes towards law students, however, resembled that for attitudes towards students of economics, that is, we found a marginally significant interaction effect on prejudice towards law students ($\Delta R^2 = .067$, $F(2, 72) = 2.72$, $p = .073$). Pro-diversity beliefs were related to more prejudice in the detrimental condition ($b = -0.709$, $SE = 0.281$, $p = .014$, $CI_{95\%} = 0.149, 1.270$) but had no effects in the control ($b = -0.012$, $SE = 0.215$, $p = .957$, $CI_{95\%} = -0.440, 0.417$) or in the instrumental condition ($b = -0.199$, $SE = 0.360$, $p = .582$, $CI_{95\%} = -0.917, 0.519$). Moreover, on feeling thermometer scores for law students the interaction between pro-diversity beliefs and instrumentality was significant ($\Delta R^2 = .084$, $F(2, 72) = 3.70$, $p = .029$). Pro-diversity beliefs were related to lower scores on the feeling thermometer in the detrimental condition ($b = -3.451$, $SE = 0.970$, $p = .001$, $CI_{95\%} = -5.384, -1.518$) but had no effects in the control ($b = -0.593$, $SE = 0.741$, $p = .426$, $CI_{95\%} = -2.070, 0.884$) or in the instrumental condition ($b = -0.259$, $SE = 1.241$, $p = .835$, $CI_{95\%} = -2.215, 2.733$). One possible explanation for theses unexpected effects of instrumentality on the relationship between pro-diversity beliefs and attitudes towards law students is that there is a high overlap in stereotype content for students of economics and law. German psychology students tend to perceive both groups as competitive and lacking a social conscience. It might therefore be that the effects for economics students generalized to law students as a secondary but similar outgroup. High intercorrelations between attitudes towards these two groups speak to this possibility also ($r = .69$, $p < .001$ and $r = .58$, $p < .001$). Students of art and philosophy, however, are perceived as very different from economics students.

References

Haddock, G., Zanna, M. P., & Esses, V. M. (1993). Assessing the structure of prejudicial attitudes: The case of attitudes toward homosexuals. *Journal of Personality and Social Psychology, 65*(6), 1105-1118. doi: 10.1037/0022-3514.65.6.1105

Hayes, A. F. (Ed.). (2013). *Methodology in the social sciences. Introduction to mediation, moderation, and conditional process analysis: A regression-based approach*. New York, London: The Guilford Press.

Honert, M. (2006). Studenten-Klischees – Die BWLer [Clichés about students – Economics students]. *Zeit Online*. Retrieved from http://www.zeit.de/campus/2006/standards/studentenklischees/bwler.

Homan, A. C., Van Knippenberg, D., Van Kleef, G. A., & De Dreu, C. K. (2007). Bridging faultlines by valuing diversity: diversity beliefs, information elaboration, and performance in diverse work groups. *Journal of Applied Psychology, 92*(5), 1189-1199. doi: 10.1037/0021-9010.92.5.1189

van Knippenberg, D., Haslam, S., & Platow, M. (2007). Unity through diversity: Value-in-diversity beliefs, work group diversity, and group identification. *Group Dynamics: theory, research, and practice, 11*(3), 207-222. doi: 10.1037/1089-2699.11.3.207
