# Supplementary material for: When good for business is not good enough: Effects of pro-diversity beliefs and instrumentality of diversity on intergroup attitudes
Source: PLoS One. 2020 Jun 1;15(6):e0234179. doi: 10.1371/journal.pone.0234179 (PMC7263624; doi:10.1371/journal.pone.0234179)
Supplement: S2 File — (PDF) [file pone.0234179.s002.pdf]

S2 File. Results for additional outgroups.

As in the pretest, we additionally addressed the question whether the moderating effect of instrumentality in diverse groups was limited to the specific outgroup participants collaborated with. In the pretest, we found some evidence for a generalization of such effects to similar outgroups. To follow up on this, we tested our predicted moderation effect of (non-) instrumentality on the relationship between pro-diversity beliefs and attitudes towards outgroups that are more similar (unemployed people) or dissimilar (politicians, wealthy people, homosexuals; for an overview of stereotype content of different social groups in Germany see Asbrock, 2010) to immigrants. We did not find any moderating effects for dissimilar outgroups (all p's > .11), with one exception: the effect of pro-diversity beliefs on general attitudes towards wealthy people was moderated by detrimental collaborations with immigrants (vs. instrumental collaborations in diverse groups; b = -1.076, SE = 0.522, p = .042, $CI_{95\%}$ = -2.111, -0.004) as well as by detrimental collaboration in non-diverse groups (vs. instrumental collaboration in diverse groups; b = -2.293, SE = 0.783, p = .004, $CI_{95\%}$ = -3.846, -0.739; overall increase in explained variance due to collaboration: ΔR^2 = .090, F(3, 99) =3.45, p = .020).

Moreover, we found a mixed pattern of results for attitudes towards the similar outgroup unemployed people. Similar to our focal outgroup immigrants, we found a significant interaction effect of negative instrumentality of interactions in diverse groups (i.e., detrimental diverse vs. instrumental diverse condition) on the relationship between pro-diversity beliefs and general attitudes toward unemployed people (b = -1.101, SE = 0.425, p = .011, $CI_{95\%}$ = -1.944, -0.259; all other p's < .05, overall increase in explained variance due to interactions: ΔR^2 = .066, F(3, 99) =2.64, p = .054). For warmth ratings towards the unemployed, we found a significant interaction effect of non-instrumentality (i.e., neutral diverse vs. instrumental diverse condition) on effects of pro-diversity beliefs (b = -0.369, SE = 0.167, p = .029, $CI_{95\%}$ = -0.700, -0.038; all other p's < .41, overall increase in explained variance due to interactions: ΔR^2 = .045, F(3, 101) =1.66, p = .182). We found a similar pattern of results for competence ratings towards the unemployed, that is a marginally significant interaction effect of non-instrumentality (i.e., neutral diverse vs. instrumental diverse condition) on effects of pro-diversity beliefs occurred (b = -0.409, SE = 0.236, p = .085, $CI_{95\%}$ = -0.877, -0.058; all other p's < .08, overall increase in explained variance due to interactions: ΔR^2 = .051, F(3, 101) =1.91, p = .132). Thus, detrimental (on general attitudes) or non-instrumental (on warmth and competence ratings) collaborations in ethnically diverse

groups reduce the positive effect of pro-diversity beliefs on attitude scores towards the similar outgroup unemployed people. To conclude, we found some evidence for a generalization effect to similar outgroups (unemployed people) but not to dissimilar outgroups (politicians, wealthy people, homosexuals), although the overall results for generalization effects to other outgroups are not fully conclusive.

References

Asbrock, F. (2010). Stereotypes of social groups in Germany in terms of warmth and competence. *Social Psychology, 41*(2), 76-81. doi: 10.1027/1864-9335/a000011

S3 Text – Study 1 – Supplemental analyses with inclusion of the covariate political orientation

We tested whether the effect of pro-diversity beliefs on attitudes towards immigrants (i.e. general attitudes, warmth and competence) was moderated by instrumentality in diverse groups (dummy coded with three variables: a) instrumental diverse as a baseline condition vs. neutral diverse, b) instrumental diverse vs. detrimental diverse, and c) instrumental diverse vs. detrimental non-diverse). We observed a significant interaction effect of negative instrumentality (i.e., instrumental diverse vs. detrimental diverse condition) on the relationship between pro-diversity beliefs and attitude scores (b = -0.959, SE = 0.405, p = .020, $CI_{95\%}$ = -1.761, -.156). However, neither non-instrumentality in diverse (neutral diverse vs. instrumental diverse condition; b = -0.593, SE = 0.487, p = .226, $CI_{95\%}$ = -1.559, .372) nor negative instrumentality in non-diverse (detrimental non-diverse vs. instrumental diverse condition; b = -0.815, SE = 0.607, p = .183, $CI_{95\%}$ = -2.020, 0.390) groups interacted with pro-diversity beliefs. Analyses of conditional effects revealed that pro-diversity beliefs had a significant positive effect on general attitude scores in all conditions (instrumental diverse: b = 2.014, SE = 0.314, p < .001, $CI_{95\%}$ = 1.391, 2.638; neutral diverse: b = 1.421, SE = 0.388, p < .001, $CI_{95\%}$ = 0.650, 2.191; detrimental diverse: b = 1.056, SE = 0.284, p < .001, $CI_{95\%}$ = 0.492, 1.620; detrimental non-diverse: b = 1.199, SE = 0.537, p = .028, $CI_{95\%}$ = 0.134, 2.264). Concerning the dependent variable warmth, we observed a significant interaction effect of negative instrumentality (i.e., detrimental diverse vs. instrumental diverse condition) on the relationship between pro-diversity beliefs and warmth (b = -0.332, SE = 0.166, p = .049, $CI_{95\%}$ = -0.661, -0.001). However, neither non-instrumentality in diverse (neutral diverse vs. instrumental diverse condition; b = -0.144, SE = 0.202, p = .478, $CI_{95\%}$ = -0.544, 0.257) nor negative instrumentality in non-diverse (detrimental non-diverse vs. instrumental diverse condition; b = -0.298, SE = 0.252, p = .240, $CI_{95\%}$ = -0.799, 0.202) groups interacted with pro-diversity beliefs. Pro-diversity beliefs had a (marginally) significant positive effect on warmth in all conditions involving collaboration in diverse groups (instrumental diverse: b = 0.558, SE = 0.129, p < .001, $CI_{95\%}$ = 0.303, 0.814; neutral diverse: b = 0.415, SE = 0.162, p = .012, $CI_{95\%}$ = 0.093, 0.736; detrimental diverse: b = 0.227, SE = 0.117, p = .055, $CI_{95\%}$ = -0.005, 0.458) but not in the detrimental non-diverse condition (b = 0.260, SE = 0.224, p = .248, $CI_{95\%}$ = -0.184, 0.704). The pattern for competence was largely comparable to the pattern for general attitudes and warmth (interaction effect negative instrumentality (detrimental diverse

vs. instrumental diverse condition) X pro-diversity beliefs: *b* = -0.349, *SE* = 0.179, *p* = .054, $CI_{95\%}$ = -0.705, 0.007; interaction effect non-instrumentality (neutral diverse vs. instrumental diverse condition) X pro-diversity beliefs: -0.300, *SE* = 0.218, *p* = .173, $CI_{95\%}$ = -0.732, 0.133; negative instrumentality in non-diverse groups (detrimental non-diverse vs. instrumental diverse condition) X pro-diversity beliefs: *b* = 0.099, *SE* = 0.273, *p* = .716, $CI_{95\%}$ = -0.442, 0.640). Pro-diversity beliefs had a (marginally) significant positive effect on competence scores in all conditions (instrumental diverse: *b* = 0.627, *SE* = 0.139, $p < .001$, $CI_{95\%}$ = 0.351, 0.903; detrimental diverse: *b* = 0.328, *SE* = 0.175, *p* = .064, $CI_{95\%}$ = -0.019, 0.675; neutral diverse: *b* = 0.278, *SE* = 0.126, *p* = .030, $CI_{95\%}$ = 0.028, 0.528; detrimental non-diverse: *b* = 0.726, *SE* = 0.242, *p* = .003, $CI_{95\%}$ = 0.247, 1.206).

S4 Text – Study 1 – Supplemental analyses without exclusion of participants that have a nationality or mother tongue other than German (*N* = 132)

We tested whether the effect of pro-diversity beliefs on attitudes towards immigrants (i.e. general attitudes, warmth and competence) was moderated by instrumentality in diverse groups (dummy coded with three variables: a) instrumental diverse as a baseline condition vs. neutral diverse, b) instrumental diverse vs. detrimental diverse, and c) instrumental diverse vs. detrimental non-diverse). We observed a significant interaction effect of negative instrumentality (i.e., instrumental diverse vs. detrimental diverse condition) on the relationship between pro-diversity beliefs and attitude scores (b = -0.797, SE = 0.394, p = .045, $CI_{95\%}$ = -1.576, -0.018). However, neither non-instrumentality in diverse (neutral diverse vs. instrumental diverse condition; b = -0.691, SE = 0.447, p = .124, $CI_{95\%}$ = -1.576, .193) nor negative instrumentality in non-diverse (detrimental non-diverse vs. instrumental diverse condition; b = -0.778, SE = 0.500, p = .122, $CI_{95\%}$ = -1.767, 0.211) groups interacted with pro-diversity beliefs. Analyses of conditional effects revealed that pro-diversity beliefs had a significant positive effect on general attitude scores in all conditions (instrumental diverse: b = 1.911, SE = 0.296, p < .001, $CI_{95\%}$ = 1.326, 2.497; neutral diverse: b = 1.220, SE = 0.335, p < .001, $CI_{95\%}$ = 0.557, 1.883; detrimental diverse: b = 1.115, SE = 0.260, p < .001, $CI_{95\%}$ = 0.601, 1.629; detrimental non-diverse: b = 1.133, SE = 0.403, p = .005, $CI_{95\%}$ = 0.336, 1.931). Concerning the dependent variable warmth, we observed a marginally significant interaction effect of negative instrumentality (i.e., detrimental diverse vs. instrumental diverse condition) on the relationship between pro-diversity beliefs and warmth (b = -0.283, SE = 0.157, p = .075, $CI_{95\%}$ = -0.594, 0.029) as well as of negative instrumentality in non-diverse groups (detrimental non-diverse vs. instrumental diverse condition; b = -0.337, SE = 0.202, p = .097, $CI_{95\%}$ = -0.736, 0.062). Non-instrumentality in diverse groups (neutral diverse vs. instrumental diverse condition; b = -0.250, SE = 0.180, p = .169, $CI_{95\%}$ = -0.606, 0.029) did not interact with pro-diversity beliefs. Pro-diversity beliefs had a significant positive effect on warmth in all conditions involving collaboration in diverse groups (instrumental diverse: b = 0.606, SE = 0.118, p < .001, $CI_{95\%}$ = 0.371, 0.840; neutral diverse: b = 0.356, SE = 0.136, p = .010, $CI_{95\%}$ = 0.087, 0.625; detrimental diverse: b = 0.323, SE = 0.104, p = .002, $CI_{95\%}$ = 0.118, 0.528) but not in the detrimental non-diverse condition (b = 0.269, SE = 0.163, p = .103, $CI_{95\%}$ = -0.055, 0.592). The pattern for competence was comparable to the pattern for general attitudes and warmth (interaction effect negative instrumentality (detrimental diverse

vs. instrumental diverse condition) X pro-diversity beliefs: $b = -0.376$, $SE = 0.170$, $p = .029$, $CI_{95\%} = -0.713, -0.038$; interaction effect non-instrumentality (neutral diverse vs. instrumental diverse condition) X pro-diversity beliefs: -0.405, $SE = 0.195$, $p = .040$, $CI_{95\%} = -0.791$, -0.019; negative instrumentality in non-diverse groups (detrimental non-diverse vs. instrumental diverse condition) X pro-diversity beliefs: $b = -0.233$, $SE = 0.219$, $p = .289$, $CI_{95\%} = -0.666, 0.200$). Pro-diversity beliefs had a significant positive effect on competence scores in all conditions (instrumental diverse: $b = 0.631$, $SE = 0.128$, $p < .001$, $CI_{95\%} = 0.377, 0.885$; detrimental diverse: $b = 0.255$, $SE = 0.112$, $p = .024$, $CI_{95\%} = 0.034, 0.477$; detrimental non-diverse: $b = 0.398$, $SE = 0.177$, $p = .026$, $CI_{95\%} = 0.048, .749$) except for the neutral diverse condition (neutral diverse: $b = 0.226$, $SE = 0.147$, $p = .127$, $CI_{95\%} = -0.065, 0.517$).

S5 Text – Study 2 – Supplemental analyses without covariate political orientation

Results of an ANOVA yielded a marginally significant main effect for pro-diversity beliefs (moral-based vs. instrumentality-based; $F(1, 299) = 3.55$, $p = .06$, $\eta^2 = .012$), a non-significant effect of instrumentality (instrumental vs. detrimental; $F(1, 299) = 1.95$, $p = .163$, $\eta^2 = .006$), and no significant interaction ($F(1, 299) = 0.12$, $p = .73$, $\eta^2 = .000$) on prejudice. Moreover, there was no significant main effect for pro-diversity beliefs (moral-based vs. instrumentality-based; $F(1, 299) = 2.37$, $p = .13$, $\eta^2 = .008$) or instrumentality (instrumental vs. detrimental; $F(1, 299) = 1.90$, $p = .17$, $\eta^2 = .006$), and no significant interaction ($F(1, 199) = 0.33$, $p = .56$, $\eta^2 = .001$) on social distance. We then tested simple main effects contrasting the factor instrumentality in dependence of pro-diversity beliefs (instrumental vs. justice-based). We neither observed an effect of the detrimental condition (compared to the instrumental condition) on prejudice in the instrumentality-based pro-diversity beliefs condition ($F(1, 299) = 1.61$, $p = .21$, $\eta^2 = .005$) nor in the morality-based pro-diversity beliefs condition ($F(1, 299) = 0.53$, $p = .47$, $\eta^2 = .002$). Moreover, we neither observed an effect of the detrimental condition (compared to the instrumental condition) on social distance in the instrumentality-based pro-diversity beliefs condition ($F(1, 299) = 2.04$, $p = .15$, $\eta^2 = .007$) nor in the morality-based pro-diversity beliefs condition ($F(1, 299) = 0.30$, $p = .58$, $\eta^2 = .001$).

S6 Text – Study 2 – Supplemental analyses without exclusion of participants with migration background (*N* = 415)

For the dependent variable prejudice, results of an ANOVA yielded a significant main effect for pro-diversity beliefs (moral vs .instrumentality-based; $F(1, 409) = 5.85$, $p = .016$, $\eta^2 = .014$), a significant effect of instrumentality (instrumental vs. detrimental; $F(1, 409) = 5.14$, $p = .024$, $\eta^2 = .012$), no significant interaction between both ($F(1, 409) = 1.13$, $p = .29$, $\eta^2 = .003$), and a significant effect of the covariate political orientation ($F(1, 409) = 174.99$, $p < .001$, $\eta^2 = .300$). For the dependent social distance, results yielded no significant main effect for pro-diversity beliefs (moral vs .instrumentality-based; $F(1, 409) = 2.16$, $p = .14$, $\eta^2 = .005$), a significant effect of instrumentality (instrumental vs. detrimental; $F(1, 409) = 14.05$, $p < .001$, $\eta^2 = .033$), no significant interaction between both ($F(1, 409) = 0.24$, $p = .62$, $\eta^2 = .001$), and a significant effect of the covariate political orientation ($F(1, 409) = 124.84$, $p < .001$, $\eta^2 = .234$). We then tested simple main effects contrasting the factor instrumentality in dependence of pro-diversity beliefs (moral vs. instrumentality-based). For prejudice, we observed a significant positive effect of the detrimental condition (compared to the instrumental condition) on prejudice in the instrumentality-based pro-diversity beliefs condition ($F (1, 409) = 5.20$, $p = .023$, partial $\eta^2 = .013$) but no effect in the morality-based pro-diversity condition ($F (1, 409) = 0.77$, $p = .38$, partial $\eta^2 = .002$). For social distance, however, we observed a significant positive effect of the detrimental condition (compared to the instrumental condition) in the instrumentality-based pro-diversity beliefs condition ($F (1, 409) = 8.43$, $p = .004$, partial $\eta^2 = .020$) as well as a significant effect in the morality-based pro-diversity condition ($F (1, 409) = 5.68$, $p = .018$, partial $\eta^2 = .014$).

S7 Text – Study 3 – Supplemental analyses with covariate political orientation

Results of an ANOVA yielded no significant main significant effect for pro-diversity beliefs (instrumental vs. justice-based; $F(1, 338) = 0.63$, $p = .43$, $\eta^2 = .002$) or instrumentality (instrumental vs. detrimental; $F(1, 338) = 1.61$, $p = .18$, $\eta^2 = .005$), and a significant interaction ($F(1, 338) = 4.10$, $p = .03$, $\eta^2 = .013$). We then tested simple main effects contrasting the factor instrumentality in dependence of pro-diversity beliefs (instrumental vs. justice-based). In line with our assumptions, we observed a significant positive effect of the detrimental condition (compared to the instrumental condition) on prejudice in the instrumentality-based pro-diversity beliefs condition (F (1, 338) = 6.51, $p = .011$, partial η^2 = .019) but no effect in the justice-based pro-diversity condition (F (1, 338) = 0.31, $p = .58$, partial $\eta^2 = .001$).

S8 Text – Study 3 – Supplemental analyses with sample without exclusion of participants with migration background and participants that took part in student exchange programs
(N = 481)

Results of an ANOVA yielded no significant main significant effect for pro-diversity beliefs (instrumental vs. justice-based; $F(1, 477) = 0.15$, $p = .90$, $\eta^2 = .000$), a significant effect of instrumentality (instrumental vs. detrimental; $F(1, 477) = 4.83$, $p = .028$, $\eta^2 = .010$), and no significant interaction ($F(1, 477) = 0.99$, $p = .32$, $\eta^2 = .002$)). We then tested simple main effects contrasting the factor instrumentality in dependence of pro-diversity beliefs (instrumental vs. justice-based). In line with our assumptions, we observed a significant positive effect of the detrimental condition (compared to the instrumental condition) on prejudice in the instrumentality-based pro-diversity beliefs condition (F (1, 477) = 5.16, p = .021, partial $\eta^2 = .011$) but no effect in the justice-based pro-diversity condition (F (1, 477) = 0.69, p = .41, partial $\eta^2 = .001$).

S9 Table – Overview of operationalizations of variables

Table 1: Constructs and respective items measured in the pretest.

Pro-diversity beliefs (university context)

- Sehr einheitliche Gruppen sind in der Regel produktiver als Gruppen mit Studierenden aus unterschiedlichen Studiengängen.
  [Homogenous groups are usually more productive than groups that are composed of students from different degree courses.]
- Wenn sich Menschen in einer Arbeitsgruppe sehr ähnlich sind, erleichtert das den Umgang mit Schwierigkeiten.
  [Having very similar people in work groups makes it easier to deal with problems.]
- In interdisziplinären Seminaren wären Studierende wesentlich produktiver als in Seminaren, die nur aus Studierenden eines einzigen Studiengangs bestehen.
  [Students would be considerably more productive in interdisciplinary courses than in courses that are composed of students from the same degree course only.]
- Wenn die unterschiedlichen Denkweisen verschiedener Studiengänge aufeinandertreffen, ist das nicht förderlich für effizientes Arbeiten.
  [Different mindsets underlying different degree courses do not facilitate efficient group work]
- Probleme können besonders gut von Gruppen gelöst werden, in denen sich Menschen aus unterschiedlichen Fachrichtungen einbringen.
  [Problems can be solved particularly well by groups involving people from different academic backgrounds.]
- Je ähnlicher sich Menschen in einer Gruppe sind, desto besser klappt die Zusammenarbeit.
  [The more similar people in a group are, the better they collaborate.]
- Von mehr Interdisziplinarität an der Uni würden alle profitieren.
  [Everyone would profit from more multidisciplinary at university.]

Pro-diversity beliefs (societal context)

- Eine Gesellschaft mit einem hohen Ausmaß an kultureller Vielfalt ist eher befähigt neue Probleme in Angriff zu nehmen.
  [A society with a high degree of cultural diversity is better able to tackle new problems.]
- Wenn Probleme auftreten, können diese besonders gut von Gruppen gelöst werden, in denen sich Menschen mit unterschiedlichen kulturellen Hintergründen einbringen.
  [Problems can best be solved by groups that include people with different cultural backgrounds.]
- Es ist besser für ein Land, wenn es eine Vielfalt unterschiedlicher Kulturen gibt.
  [It is better for a country if there exists a variety of different cultures.]
- Wenn sich die Menschen in einer Gesellschaft sehr ähnlich sind, erleichtert das den Umgang mit neuen Problemen.
  [It makes it easier to deal with new problems when people in a society are very similar to each other.]
- Kulturell vielfältige Gruppen sind in der Regel produktiver als sehr einheitliche Gruppen.
  [Culturally diverse groups are usually more productive than culturally homogenous groups.]

Manipulation check: Instrumentality of interactions

- Wie hilfreich waren die Beiträge Ihrer Teampartner zur Gesamtleistung?
  [How helpful were your team partners' contributions to your team's performance?]
- Wie nützlich erschien Ihnen die Zusammenarbeit mit Ihren Teampartnern?
  [How useful did you perceive the collaboration with your team partners?]

Prejudice

- [group] und Psychologen haben sehr unterschiedliche Wertvorstellungen.
  [(group) and psychologists have very different values.]
- Ein Psychologie-Studium bietet eine wesentlich bessere Ausbildung als ein [group]-Studium.
  [Studies of psychology offer a much better training than studies of (group).]
- Ich mag [group].
  [I like (group).]
- Die Universitäts-Verwaltung sollte lieber mehr Geld in den Fachbereich Psychologie investieren statt in die Fakultät für [group].
  [The university administration should invest more money in the department of psychology than in the department of (group).]
- Das [group]-Studium genießt immer noch ein zu positives Bild in den Medien.
  [Studies in (group) are still portrayed too positively in the media.]
- Die meisten [group] sind vor allem an Geld und Karriere interessiert.
  [Most (group) are primarily interested in earning money and making a career.]
- Ich würde ungern mit einem [group] in eine WG ziehen.
  [I would be reluctant to move into a shared apartment with a (group).]
- Es sollte viel [group] geben.
  [There should be many (group).]
- Ich halte [group] für interessante Menschen.
  [I think (group) are interesting people.]

Feeling thermometer

- Wie würden Sie allgemein Ihre Gefühle gegenüber [group] bezeichnen?
  [In general, how would you rate your feelings towards (group)?]

Intergroup contact

- Wie viel Kontakt haben Sie in ihrem Freundes- und Bekanntenkreis zu [group]?
  [How much contact do you have with (group) in your circle of your friends and acquaintances?]

Political orientation

- Wenn Sie an Ihre eigenen politischen Ansichten denken, würden Sie sich selbst als eher links oder eher rechts bezeichnen?
  [Thinking of your political attitudes, would you consider yourself more left or more right?]

Table 2: Constructs and respective items measured in Study 1.

Pro-diversity beliefs

- Eine Gesellschaft mit einem hohen Ausmaß an kultureller Vielfalt ist eher befähigt neue Probleme in Angriff zu nehmen.
  [A society with a high degree of cultural diversity is better able to tackle new problems.]
- Wenn Probleme auftreten, können diese besonders gut von Gruppen gelöst werden, in denen sich Menschen mit unterschiedlichen kulturellen Hintergründen einbringen.
  [Problems can best be solved by groups that include people with different cultural backgrounds.]
- Es ist besser für ein Land, wenn es eine Vielfalt unterschiedlicher Kulturen gibt.
  [It is better for a country if there exists a variety of different cultures.]
- Wenn sich die Menschen in einer Gesellschaft sehr ähnlich sind, erleichtert das den Umgang mit neuen Problemen..
  [It makes it easier to deal with new problems when people in a society are very similar to each other.]
- Kulturell vielfältige Gruppen sind in der Regel produktiver als sehr einheitliche Gruppen.
  [Culturally diverse groups are usually more productive than culturally homogenous groups.]
- Ich schätze die Vielfalt in Deutschland, weil sie dem Land einen Nutzen bringt.
  [I value cultural diversity in Germany because it benefits the country.]
- Kulturelle Vielfalt bringt Deutschland voran und sollte daher gefördert werden.
  [Cultural diversity helps Germany get ahead and should therefore be supported.]

General intergroup attitudes

- Wie würden Sie [group] allgemein bewerten?
  [In general, how would you evaluate (group)?]

Warmth

Please rate [group] with regard to the following attributes,

- freundlich
  [friendly]
- sympathisch
  [likeable]
- warm
  [warm]

Competence

- abhängig
  [dependent]
- kompetent
  [competent]

- leistungsfähig
  [efficient]

Distractor items: Climate change

- Der Klimawandel bedroht die Existenz der Menschheit.
  [Climate change threatens the existence of mankind.]
- Für den Klimawandel ist vor allem der Mensch verantwortlich.
  [It is humans that are primarily responsible for climate change.]
- Der Klimawandel wird in der Öffentlichkeit übertrieben dargestellt.
  [Climate change is exaggerated in public discourse.]
- Wissenschaft und Technik werden die Umwelt- und Klimaprobleme lösen, ohne dass wir unsere Lebensweise ändern müssen.
  [Science and technical developments will solve environmental and climate change related problems, without us having to change our way of life.]
- Das Thema Klimawandel wird größer gemacht als es ist.
  [The topic of climate change is being made out to be bigger than it actually is.]
- Die Folgen des Klimawandels werden nicht so dramatisch aussehen, wie es uns die Experten weismachen wollen.
  [Consequences of climate change won't be as dramatic as experts want to make us believe.]
- Der Klimawandel lässt sich nicht mehr aufhalten.
  [Climate change cannot be stopped.]
- Ich mache mir keine Sorgen um den Klimawandel.
  [I do not worry about climate change.]

Distractor items: Demographic change

- Ich halte es für problematisch, dass es in Deutschland bald immer mehr ältere und weniger junge Menschen geben wird.
  [I think it is a problem that there will be more and more elderly and fewer and fewer young people in Germany.]
- Das Verhältnis zwischen jungen und alten Menschen in Deutschland ist angespannt.
  [The relationship between young and old people in Germany is tense.]
- Das Verhältnis zwischen jungen und alten Menschen in Deutschland wird sich zukünftig verschlechtern.
  [The relationship between young and old people in Germany will get worse in the future.]
- Das gesetzliche Rentenalter sollte weiter erhöht werden.
  [The statutory retirement age should be increased.]
- Altersarmut wird in Zukunft zu einem der wichtigsten Themen in Deutschland werden.
  [Poverty among the elderly will become one of the most important topics for Germany in the future.]
- Alte Menschen sollten bereit sein, finanziell zurück zu stecken.
  [The elderly should be willing to aim lower with regard to their finances.]
- Junge Menschen sind undankbar.
  [Young people are ungrateful.]

Distractor items: Financial crisis

- Ich glaube, dass die Politik alles tut, um den Menschen, die unter der Finanzkrise leiden, zu helfen.
  [I believe that politicians are doing everything they can to help those that suffer from the financial crisis.]
- Ich habe großes Vertrauen in die Arbeit der Europäischen Zentralbank (EZB).
  [I have great trust in the work of the European Central Bank (ECB).]
- Ich mache mir Sorgen um meine Ersparnisse.
  [I worry about my savings.]
- Die Auswirkungen der Finanzkrise werden wir noch mehrere Jahre zu spüren bekommen.
  [We will still feel the effects of the financial crisis in years to come.]
- Banken sollten stärker kontrolliert werden.
  [Banks should be controlled more strictly.]
- Ich sehe keinen Sinn in der Rettung des Euros.
  [I do not see any value in saving the Euro.]
- Das Krisenmanagement der Europäischen Union nach der Finanzkrise war im Großen und Ganzen ein Erfolg.
  [In general, the crisis management of the European Union after the financial crisis was a success.]
- Ich mache mir Sorgen um meine Altersvorsorge.
  [I worry about my retirement provision.]

Distractor items: Personality

- Ich fühle mich wohl unter Menschen.
  [I feel comfortable being among other people.]
- Ich finde leicht Freunde.
  [I make friends easily.]
- Ich habe eine reiche Fantasie.
  [I have a vivid imagination.]
- Ich suche selten nach der tieferen Bedeutung in etwas.
  [I rarely look for a deeper meaning in things.]
- Ich glaube, dass andere generell gute Absichten haben.
  [I believe that others have good intentions.]
- Ich möchte, dass sich andere wohlfühlen.
  [I want others to feel comfortable.]
- Ich achte auf Details.
  [I pay attention to details.]
- Ich mache nur so viel Arbeit, wie nötig ist.
  [I do just enough work to get by.]
- Ich gerate leicht in Panik.
  [I panic easily.]
- Ich bin zufrieden mit mir.
  [I am comfortable with myself.]
- Ich akzeptiere Menschen so, wie sie sind.

[I accept people as they are.]

- Ich bin immer gut vorbereitet.
  [I am always well prepared.]
- Ich habe häufig Stimmungsschwankungen.
  [I have frequent mood swings.]
- Ich denke, dass Kunst wichtig ist.
  [I believe in the importance of arts.]

Political orientation

- Viele Leute verwenden die Begriffe "links" und "rechts", wenn es darum geht, unterschiedliche politische Einstellungen zu kennzeichnen. Wenn Sie an Ihre eigenen politischen Ansichten denken, wie würden Sie sich selbst einschätzen?
  [Many people use the terms “left” and “right” to describe political attitudes. Thinking of your political attitudes, how would you consider yourself?]

Table 3: Constructs and respective items measured in Study 2.

Prejudice

- Es gibt zu viele Flüchtlinge in Deutschland.
  [There are too many refugees in Germany.]
- Flüchtlinge sind eine Belastung für das Sozialsystem.
  [Refugees are a burden for the social welfare system.]
- Die meisten Flüchtlinge sind vor allem an Geld interessiert.
  [Most refugees are primarily interested in money.]
- Deutschland braucht eine strengere Asylpolitik.
  [Germany needs stricter asylum policies.]

Social distance

- Ich würde versuchen, mit den Bewohnern der Flüchtlingsunterkunft in Kontakt zu kommen.
  [I would try to get into contact with the people living in the refugee home.]
- Ich wäre bereit, Flüchtlinge als Gäste zu mir nach Hause einzuladen.
  [I would be willing to invite refugees to my home.]
- Ich wäre bereit, Flüchtlinge in der Flüchtlingsunterkunft zu besuchen.
  [I would be willing to visit refugees in a refugee home.]
- Ich würde nur ungern in die Nähe der Flüchtlingsunterkunft ziehen.
  [I would be reluctant to move to a neighborhood with a refugee home.]

Manipulation check: Instrumentality of refugees

- Die Schwalmtaler Bürger haben von der Flüchtlingsunterkunft profitiert.
  [The citizens of Schwalmtal profited from the refugee home.]
- Die Flüchtlingsunterkunft ist ein Gewinn für Schwalmtal.
  [The refugee home is a benefit to Schwalmtal.]

Political orientation

- Viele Leute verwenden die Begriffe „links“ und „rechts“, wenn es darum geht, unterschiedliche politische Einstellungen zu kennzeichnen. Wenn Sie an Ihre eigenen politischen Ansichten denken, wie würden Sie sich selbst einschätzen?
  [Many people use the terms “left” and “right” to describe political attitudes. Thinking of your political attitudes, how would you consider yourself?]]

Distractor items: Contact with refugees

- Bitte geben Sie an, wie häufig Sie Kontakt mit Flüchtlingen in den unterschiedlichen Bereichen haben:
  In der Schule, im Studium oder im Beruf

[Please indicate how often you have contact with refugees in different domains: At school, at university or at work]

- In Ihrer unmittelbaren Wohnnachbarschaft
  [In your direct neighborhood]
- In Ihrem Freundeskreis
  [among your circle of friends]

Distractor items: Attitudes towards political reactions to the refugee crisis

- Ich finde die Bundesregierung macht einen guten Job im Umgang mit der Flüchtlingsproblematik.
  [I think that the government is doing a good job in dealing with the refugee crisis.]
- Ich finde die Bundeskanzlerin macht einen guten Job im Umgang mit der Flüchtlingsproblematik.
  [I think that the chancellor is doing a good job in dealing with the refugee crisis.]]
- Ich finde die EU macht einen guten Job im Umgang mit der Flüchtlingsproblematik.
  [I think that the EU is doing a good job in dealing with the refugee crisis.]]

Distractor items: Interest in and knowledge of the topics asylum and refugee homes

- Wie groß ist Ihr Interesse an den Themen Flucht und Asyl?
  [How interested are you in the topics fleeing and asylum?]
- Wie viele neue Informationen beinhaltete dieser Text für Sie?
  [How much information given in the text was new to you?]
- Wie groß ist Ihr Interesse an Fragestellungen zum den Themen Unterbringung und Integration von Flüchtlingen?
  [How interested are you in the topics housing and integration of refugees?]
- Wie viele neue Informationen beinhaltete dieser Text für Sie?
  [How much information given in the text was new to you?]

Attention check

- Wenn ich die Fragen aufmerksam lese, kreuze ich hier eine 5 an.
  [If I ready this carefully I will check the field ‘5’.]

Table 4: Manipulation texts used in Study 2.

moral-based pro-diversity beliefs	Instrumentality-based pro-diversity beliefs
[…] Experten und Politiker weisen in diesem Zusammenhang darauf hin, dass es als moralische Verpflichtung Deutschlands gilt, sich für die Flüchtlinge einzusetzen. Deutschland bekennt sich zur Allgemeinen Erklärung der Menschenrechte, aus der hervorgeht, dass jeder Mensch das Recht auf Leben, Freiheit und Sicherheit der eigenen Person hat.<br><br>[Experts and politicians have pointed out that Germany has a moral obligation to support refugees. Germany has ratified the Universal Declaration of Human Rights and is hence bound to protect the rights to life, liberty, and personal safety of every human being.]	[…] Experten und Politiker weisen in diesem Zusammenhang darauf hin, dass Deutschland einen hohen Nutzen daraus ziehen kann, wenn es sich für Flüchtlinge einsetzt. Die Vergangenheit hat gezeigt, dass Deutschlands Wirtschaft von Zuwanderung und Vielfalt profitiert. Ehemalige Flüchtlinge kompensieren an einigen Stellen den Fachkräftemangel und leisten einen wichtigen Beitrag zum Erfolg deutscher Unternehmen. Die unterschiedlichen Perspektiven, die Menschen aus dem Ausland mitbringen, schaffen innovative Lösungen für Probleme und öffnen neue Märkte.<br><br>[Experts and politicians have pointed out that Germany can profit from supporting refugees. The past has shown that the German economy benefits from immigration and diversity. Former refugees help to compensate for the lack of a skilled workforce and contribute to the success of German companies. Different perspectives brought in by people from abroad generate innovative solutions for problems and create new market opportunities.]
**instrumental**	**detrimental**
[…] Die Geschichte der Flüchtlingsunterkunft in Schwalmtal ist eine Erfolgsgeschichte“, erklärt Bürgermeister Markus Piesch. „Man kann wirklich sagen, dass die Schwalmtaler Bürger von der Unterkunft profitieren.“ Dies zeige sich beispielsweise darin, dass im Fußballverein mittlerweile ca. ein Viertel der Spieler Flüchtlinge sind und es ein großes Angebot an Tandem-Sprachkursen gibt, in denen die Schwalmtaler, die Sprachen der Flüchtlinge lernen können. Einige der Flüchtlinge sind nach der Anerkennung ihrer	[…] „Die Geschichte der Flüchtlingsunterkunft in Schwalmtal ist alles andere als eine Erfolgsgeschichte“, erklärt Bürgermeister Markus Piesch. „Man muss leider sagen, dass die Unterkunft für die Schwalmtaler Bürger eine Belastung darstellt.“ Dies zeige sich beispielsweise darin, dass die Preise von angrenzenden Immobilien seit der Einrichtung der Unterkunft gesunken sind. Auch kostete eine Sanierung der Unterkunft im Jahr 2013 die Gemeinde Schwalmtal viel Geld, das nun an anderen Stellen fehle. Die Hoffnung klein- und

Asylanträge in Schwalmtal geblieben und haben durch ihre Arbeit als Krankenpfleger und Handwerker einige kleine und mittlere Betriebe vor Ort gestärkt. Andere Flüchtlinge konnten ihren Ingenieursabschluss anerkennen lassen und arbeiten jetzt bei einem großen Nahrungsmittelhersteller als Mechatroniker. Missverständnisse und Konflikte zwischen Schwalmtalern und Flüchtlingen hat es nur sehr selten gegeben.

[“The history of refugee accommodation in Schwalmtal is a success story,” explains Mayor Markus Piesch. "One can truly say that the citizens of Schwalmtal benefit from the accommodation." This is demonstrated, for example, by the fact that about a quarter of the football club consists of refugees, and that there is a wide range of tandem language courses in which the citizens of Schwalmtal learn the languages of the refugees. Some of the refugees have remained in Schwalmtal after the recognition of their asylum applications and have strengthened a number of local small and medium-sized enterprises through their work as nurses and craftsmen. Other refugees have received their engineering degree and are now working as mechatronics engineer for a major food manufacturer. Misunderstandings and conflicts between citizens of Schwalmtal and refugees have been very rare.]

mittelständischer Betriebe auf ausbildungswilligen Nachwuchs hat sich nicht erfüllt: Viele Flüchtlinge ziehen nach der Anerkennung ihrer Asylanträge einfache Hilfsarbeiten vor. Einen Austausch zwischen Schwalmtalern und Flüchtlingen, zum Beispiel beim örtlichen Fußballverein, gibt es nur selten. Zudem sind die wenigen Begegnungen zwischen Schwalmtalern und Flüchtlingen oft von Missverständnissen und Konflikten geprägt.

[“The history of refugee accommodation in Schwalmtal is anything but a success story," explains Mayor Markus Piesch. "One has to say, unfortunately, that the accommodation is a burden for the citizens.” This is demonstrated, for example, by the fact that the prices of adjacent properties have fallen since the establishment of the accommodation. Also, a renovation of the property in 2013, has cost the municipality Schwalmtal a lot of money – money which is now lacking elsewhere. The hope by small and medium-sized companies that refugees would be willing to fill job training vacancies has not been fulfilled: After acceptance of their asylum applications most refugees prefer simple occasional jobs. Exchanges between citizens of Schwalmtal and refugees, for example, at the local football club, very rarely happen. In addition, the few encounters between citizens of Schwalmtal and refugees are often marked by misunderstandings and conflicts.]

Table 5: Constructs and respective items measured in Study 3.

Prejudice

- Ausländische Studierende sind fleißig.
  [Foreign exchange students are hard-working.]
- An deutschen Hochschulen gibt es zu viele ausländische Studierende.
  [There are too many foreign students at German universities.]
- Ausländische Studierende sind freundlich und offen.
  [Foreign students are friendly and open-minded.]
- Die meisten ausländischen Studierenden genießen eine gute Bildung ohne eine Gegenleistung zu erbringen.
  [Most foreign students profit from good education but do not provide anything in return.]
- Ich würde ungern an einer Hochschule studieren, an der es sehr viele ausländische Studierende gibt.
  [I would be reluctant to study at a university that includes a lot of foreign students.]

Manipulation check: Instrumentality of exchange students

- Je internationaler die Hochschulen in Deutschland sind, desto höher ist die Qualität der Forschung..
  [The more international German universities are, the higher the quality of research.]
- Je internationaler die Hochschulen in Deutschland sind, desto zufriedener sind die Studierenden.
  [The more international German universities are, the more satisfied the students.]
- Deutsche Hochschulen profitieren von ausländischen Studierenden.
  [German universities profit from international students.]

Political orientation

- Viele Leute verwenden die Begriffe „links" und „rechts", wenn es darum geht, unterschiedliche politische Einstellungen zu kennzeichnen. Wenn Sie an Ihre eigenen politischen Ansichten denken, wie würden Sie sich selbst einschätzen?
  [Many people use the terms "left" and "right" to describe political attitudes. Thinking of your political attitudes, how would you consider yourself?]]

Distractor items: Contact with exchange students

- Bitte geben Sie an, wie häufig Sie Kontakt mit ausländischen Studierenden in den unterschiedlichen Bereichen haben:
  In der Schule, im Studium oder im Beruf
  [Please indicate how often you have contact with foreign exchange students in different domains: At school, at university or at work]
- In Ihrer unmittelbaren Wohnnachbarschaft
  [In your direct neighborhood]

- In Ihrem Freundeskreis
  [Among your circle of friends]

Distractor items: General attitudes towards and knowledge of student exchange programs

- Über die unterschiedlichen Möglichkeiten, die es bei internationalen Studierendenprogrammen gibt, fühle ich mich ausreichend informiert.
  [I feel that I have sufficient information about the opportunities of international study programs.]
- Internationale Studierendenprogramme, wie z. B. das Erasmus-Programm, halte ich für sehr sinnvoll.
  [I think that international study programs, such as the Erasmus program, are useful.]
- Die Angebote sollte noch weiter ausgebaut werden, damit noch mehr Interessierte von internationalen Studierendenprogrammen profitieren können.
  [Existing offers should be expanded to allow even more interested people to profit from international study programs.]

Distractor items: Own experiences with international student programs

- Haben Sie schon einmal im Rahmen Ihres Studiums einen Auslandsaufenthalt absolviert
  [Did you spend some time abroad as part of your studies?]
- Wenn ja, in welchem Land?
  [If so, in which country?]
- Welche Schulnote würden Sie Ihrem Aufenthalt geben?
  [, How would you rate your stay, using school grades?]
- Welche Schulnote würden Sie der Qualität des Angebots für ausländische Studierende an Ihrer Universität geben?
  [How would you rate the quality of opportunities for foreign students provided by the university, sing school grades?]
- Wenn sich mir die Möglichkeit zu einem Studium im Ausland bieten würde, würde ich diese Möglichkeit (noch einmal) nutzen.
  [If I had the opportunity to study abroad, I would take it (once more).]

Attention check

- Wenn Sie dies hier lesen, wählen Sie bitte eine 0 aus.
  [If you read this please select ‘0’.]
- Wenn Sie dies hier lesen, wählen Sie bitte eine 5 aus.
  [If you read this please select ‘5’.]

Table 6: Manipulation texts used in Study 3.

justice-based pro-diversity beliefs	Instrumentality-based pro-diversity beliefs
[…] Prof. Dr. Johanna Wanka, Bundesministerin für Bildung und Forschung betont dabei, dass „Deutschland ausländischen Studierenden das Studium in Deutschland ermöglicht, weil es sich gegenüber seinen EU-Partnern in der Verantwortung sieht.“ und ergänzt, „Nur wenn alle Länder bei Studierendenaustauschen kooperieren, kann die EU ihren BügerInnen einen gerechten Zugang zu Bildung und Chancen ermöglichen.“	[…] Prof. Dr. Johanna Wanka, Bundesministerin für Bildung und Forschung betont dabei, dass „die Aufnahme ausländischer Studierender für die deutschen Hochschulen einen Nutzen mit sich bringt.“ und ergänzt, „Wir gehen davon aus, dass Hochschulen und deren Angehörige von Internationalisierung profitieren -beispielweise, weil unterschiedliche Perspektiven Forschungs- und Lehrprozesse anregen und verbessern können.“
[Prof. Wanka, the German Minister for Education and research, emphasizes that Germany allows foreign students to study at German universities because Germany is bound to support its EU partners. She claims: “Only if all countries cooperate properly with regard to exchange study programs are we able to grant all EU citizens fair access to education.”]	[Prof. Wanka, the German Minister for Education and research, emphasizes that German universities profit from receiving foreign students. She claims: “We believe that universities and members of universities profit from internationalization – for example because the diversity of perspectives can improve teaching and research.”]
**instrumental**	**detrimental**
[…] Eine Auswertung der Ergebnisse aller deutschen Universitäten ergab nach Kontrolle anderer relevanter Faktoren (z. B. Standort und Größe der Universität), dass sich die Präsenz ausländischer Studierender positiv auf die Qualität der Forschung und die Zufriedenheit der Studierenden an deutschen Hochschulen auswirkt.	[…] Eine Auswertung der Ergebnisse aller deutschen Universitäten ergab nach Kontrolle anderer relevanter Faktoren (z. B. Standort und Größe der Universität), dass sich die Präsenz ausländischer Studierender negativ auf die Qualität der Forschung und die Zufriedenheit der Studierenden an deutschen Hochschulen auswirkt.
[The analysis of results for all German universities indicates that – while controlling for other relevant factors (e.g., location, size of university) – the presence of international students has a positive effect on the quality of research as well as on student satisfaction.]	[The analysis of results for all German universities indicates that – while controlling for other relevant factors (e.g., location, size of university) – the presence of international students has a negative effect on the quality of research as well as on student satisfaction.]

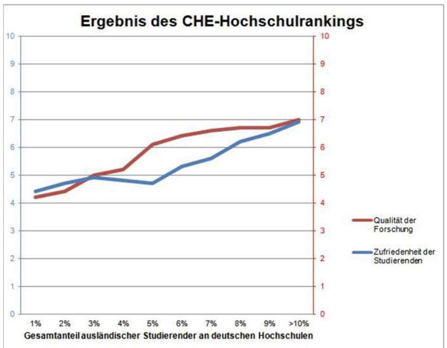

Qualität der Forschung: Der Index ergibt sich aus Zahl der wissenschaftlichen Veröffentlichungen (z.T. gewichtet) pro Wissenschaftler/-in in einem 3-Jahres-Zeitraum sowie Drittmittel aus Industrie, Stiftungen, DFG usw. (in Tausend Euro) im Verhältnis zur Zahl der Wissenschaftlerinnen und Wissenschaftler.
Zufriedenheit der Studierenden: Der Index ergibt sich aus dem Mittelwert der verschiedenen Einzelfragen zur Bewertung der Studiensituation durch die Studierenden auf den Skalen von 1 (sehr schlecht) bis 10 (ausgezeichnet) .

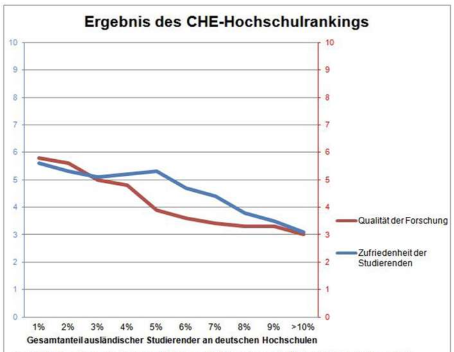

Qualität der Forschung: Der Index ergibt sich aus Zahl der wissenschaftlichen Veröffentlichungen (z.T. gewichtet) pro Wissenschaftler/-in in einem 3-Jahres-Zeitraum sowie Drittmittel aus Industrie, Stiftungen, DFG usw. (in Tausend Euro) im Verhältnis zur Zahl der Wissenschaftlerinnen und Wissenschaftler.
Zufriedenheit der Studierenden: Der Index ergibt sich aus dem Mittelwert der verschiedenen Einzelfragen zur Bewertung der Studiensituation durch die Studierenden auf den Skalen von 1 (sehr schlecht) bis 10 (ausgezeichnet) .

X

Descriptive statistics for variables in the pretest and Study 1

Table S7: Means, standard deviations, and intercorrelations of measures of the pretest.

	n	*M*	*SD*	2	3	4	5	6	7	8	9	10
1 perceived instrumentality	52	3.31	1.16	.08	.06	.03	.21	.01	-.04	-.11	.06	-.02
2 pro-diversity beliefs	78	3.49	0.45		-.06	.11	-.18	-.21	-.03	-.22	.09	-.06
3 prej. tw. economics students	78	2.78	0.55			.69***	.22	.17	-.52***	-.32**	.18	-.02
4 prej. tw. law students	78	2.64	0.61				.31**	.22	-.23*	-.56***	.12	-.02
5 prej. tw. philosophy students	78	2.20	0.54					.66***	.12	.16	-.49***	-.39**
6 prej. tw. art students	78	1.98	0.45						.03	.12	-.31**	-.64***
7 feel thermometer economics students	78	6.19	2.06							.58***	-.13	.09
8 feel thermometer. law students	78	6.10	2.18								-.22	-.03
9 feel thermometer philosophy students	78	7.13	2.19									.55***

10 feel thermometer. art students	78	8.03	1.93

Note. prej. = prejudice; * $p < .05$, ** $p < .01$, *** $p < .001$

Table S8: Means, standard deviations, and intercorrelations of additional measures of Study 1

	M	*SD*	2	3	4	5	6	7	8	9	10	11	12	13	14	15	16
1 pro-diversity beliefs	3.79	0.82	.66**	.19*	.31**	.19	.39**	.49**	-.04	.15	.12	.24*	.47**	.18	.20*	.03	.29**
2 feel thermometer immigrants	6.31	1.89		.18	.49**	.35**	.54**	.63**	.03	.32**	.13	.27**	.63**	.18	.28**	.11	.40**
3 feel thermometer politicians	4.26	1.86			.16	.48**	.11	.12	.49**	.02	.42**	.03	.20*	.50**	.17	.22*	.12
4 feel thermometer unemployed persons	5.21	1.56				.14	.26**	.21*	-.04	.55**	.11	.03	.29**	.06	.61**	-.10	.23*
5 feel thermometer the rich	4.98	1.88					.23*	.11	.22*	-.02	.55**	.06	.11	.22*	.15	.34**	.04

6 feel thermometer homosexuals	7.22	1.83	.41**	-.12	.19	-.03	.72**	.41**	.18	.15	.29**	.63**
7 warmth immigrants	3.31	0.68		.06	.40**	.16	.51**	.72**	.23*	.17	.28**	.46**
8 warmth politicians	2.57	0.63			.07	.54**	-.06	.05	.35**	.13	.07	-.04
9 warmth unemployed persons	3.05	0.49				.14	.22*	.40**	.11	.54**	.03	.35**
10 warmth the rich	2.60	0.65					-.05	.15	.31**	.24*	.24*	-.05
11 warmth homosexuals	3.71	0.68						.35**	.15	.02	.37**	.670**
12 competence immigrants	3.23	0.73							.31**	.37**	.22*	.53**

13 competence politicians	3.23	0.80	.15	.27**	.15
14 competence unemployed persons	2.85	0.70		-.02	.26**
15 competence the rich	3.34	0.78			.27**
16 competence homosexuals	3.43	0.67			

Note. $N = 109$; * $p < .05$, ** $p < .01$, *** $p < .001$
