# Supplementary material for: When good for business is not good enough: Effects of pro-diversity beliefs and instrumentality of diversity on intergroup attitudes
Source: PLoS One. 2020 Jun 1;15(6):e0234179. doi: 10.1371/journal.pone.0234179 (PMC7263624; doi:10.1371/journal.pone.0234179)
Supplement: S3 File — (PDF) [file pone.0234179.s003.pdf]

S3 File. Questionnaire items and texts for all studies.

Table 1: Constructs and respective items measured in the pretest.

Pro-diversity beliefs (university context)

- Sehr einheitliche Gruppen sind in der Regel produktiver als Gruppen mit Studierenden aus unterschiedlichen Studiengängen.
  [Homogenous groups are usually more productive than groups that are composed of students from different degree courses.]
- Wenn sich Menschen in einer Arbeitsgruppe sehr ähnlich sind, erleichtert das den Umgang mit Schwierigkeiten.
  [Having very similar people in work groups makes it easier to deal with problems.]
- In interdisziplinären Seminaren wären Studierende wesentlich produktiver als in Seminaren, die nur aus Studierenden eines einzigen Studiengangs bestehen.
  [Students would be considerably more productive in interdisciplinary courses than in courses that are composed of students from the same degree course only.]
- Wenn die unterschiedlichen Denkweisen verschiedener Studiengänge aufeinandertreffen, ist das nicht förderlich für effizientes Arbeiten.
  [Different mindsets underlying different degree courses do not facilitate efficient group work]
- Probleme können besonders gut von Gruppen gelöst werden, in denen sich Menschen aus unterschiedlichen Fachrichtungen einbringen.
  [Problems can be solved particularly well by groups involving people from different academic backgrounds.]
- Je ähnlicher sich Menschen in einer Gruppe sind, desto besser klappt die Zusammenarbeit.
  [The more similar people in a group are, the better they collaborate.]
- Von mehr Interdisziplinarität an der Uni würden alle profitieren.
  [Everyone would profit from more multidisciplinary at university.]

Pro-diversity beliefs (societal context)

- Eine Gesellschaft mit einem hohen Ausmaß an kultureller Vielfalt ist eher befähigt neue Probleme in Angriff zu nehmen.
  [A society with a high degree of cultural diversity is better able to tackle new problems.]
- Wenn Probleme auftreten, können diese besonders gut von Gruppen gelöst werden, in denen sich Menschen mit unterschiedlichen kulturellen Hintergründen einbringen.
  [Problems can best be solved by groups that include people with different cultural backgrounds.]
- Es ist besser für ein Land, wenn es eine Vielfalt unterschiedlicher Kulturen gibt.
  [It is better for a country if there exists a variety of different cultures.]
- Wenn sich die Menschen in einer Gesellschaft sehr ähnlich sind, erleichtert das den Umgang mit neuen Problemen.
  [It makes it easier to deal with new problems when people in a society are very similar to each other.]

- Kulturell vielfältige Gruppen sind in der Regel produktiver als sehr einheitliche Gruppen.
  [Culturally diverse groups are usually more productive than culturally homogenous groups.]

Manipulation check: Instrumentality of interactions

- Wie hilfreich waren die Beiträge Ihrer Teampartner zur Gesamtleistung?
  [How helpful were your team partners' contributions to your team's performance?]
- Wie nützlich erschien Ihnen die Zusammenarbeit mit Ihren Teampartnern?
  [How useful did you perceive the collaboration with your team partners?]

Prejudice

- [group] und Psychologen haben sehr unterschiedliche Wertvorstellungen.
  [(group) and psychologists have very different values.]
- Ein Psychologie-Studium bietet eine wesentlich bessere Ausbildung als ein [group]-Studium.
  [Studies of psychology offer a much better training than studies of (group).]
- Ich mag [group].
  [I like (group).]
- Die Universitäts-Verwaltung sollte lieber mehr Geld in den Fachbereich Psychologie investieren statt in die Fakultät für [group].
  [The university administration should invest more money in the department of psychology than in the department of (group).]
- Das [group]-Studium genießt immer noch ein zu positives Bild in den Medien.
  [Studies in (group) are still portrayed too positively in the media.]
- Die meisten [group] sind vor allem an Geld und Karriere interessiert.
  [Most (group) are primarily interested in earning money and making a career.]
- Ich würde ungern mit einem [group] in eine WG ziehen.
  [I would be reluctant to move into a shared apartment with a (group).]
- Es sollte viel [group] geben.
  [There should be many (group).]
- Ich halte [group] für interessante Menschen.
  [I think (group) are interesting people.]

Feeling thermometer

- Wie würden Sie allgemein Ihre Gefühle gegenüber [group] bezeichnen?
  [In general, how would you rate your feelings towards (group)?]

Intergroup contact

- Wie viel Kontakt haben Sie in ihrem Freundes- und Bekanntenkreis zu [group]?
  [How much contact do you have with (group) in your circle of your friends and acquaintances?]

Political orientation

- Wenn Sie an Ihre eigenen politischen Ansichten denken, würden Sie sich selbst als eher links oder eher rechts bezeichnen?
  [Thinking of your political attitudes, would you consider yourself more left or more right?]

Table 2: Constructs and respective items measured in Study 1.

Pro-diversity beliefs

- Eine Gesellschaft mit einem hohen Ausmaß an kultureller Vielfalt ist eher befähigt neue Probleme in Angriff zu nehmen.
  [A society with a high degree of cultural diversity is better able to tackle new problems.]
- Wenn Probleme auftreten, können diese besonders gut von Gruppen gelöst werden, in denen sich Menschen mit unterschiedlichen kulturellen Hintergründen einbringen.
  [Problems can best be solved by groups that include people with different cultural backgrounds.]
- Es ist besser für ein Land, wenn es eine Vielfalt unterschiedlicher Kulturen gibt.
  [It is better for a country if there exists a variety of different cultures.]
- Wenn sich die Menschen in einer Gesellschaft sehr ähnlich sind, erleichtert das den Umgang mit neuen Problemen..
  [It makes it easier to deal with new problems when people in a society are very similar to each other.]
- Kulturell vielfältige Gruppen sind in der Regel produktiver als sehr einheitliche Gruppen.
  [Culturally diverse groups are usually more productive than culturally homogenous groups.]
- Ich schätze die Vielfalt in Deutschland, weil sie dem Land einen Nutzen bringt.
  [I value cultural diversity in Germany because it benefits the country.]
- Kulturelle Vielfalt bringt Deutschland voran und sollte daher gefördert werden.
  [Cultural diversity helps Germany get ahead and should therefore be supported.]

General intergroup attitudes

- Wie würden Sie [group] allgemein bewerten?
  [In general, how would you evaluate (group)?]

Warmth

Please rate [group] with regard to the following attributes,

- freundlich
  [friendly]
- sympathisch
  [likeable]
- warm
  [warm]

Competence

- abhängig
  [dependent]
- kompetent
  [competent]

- leistungsfähig
  [efficient]

Distractor items: Climate change

- Der Klimawandel bedroht die Existenz der Menschheit.
  [Climate change threatens the existence of mankind.]
- Für den Klimawandel ist vor allem der Mensch verantwortlich.
  [It is humans that are primarily responsible for climate change.]
- Der Klimawandel wird in der Öffentlichkeit übertrieben dargestellt.
  [Climate change is exaggerated in public discourse.]
- Wissenschaft und Technik werden die Umwelt- und Klimaprobleme lösen, ohne dass wir unsere Lebensweise ändern müssen.
  [Science and technical developments will solve environmental and climate change related problems, without us having to change our way of life.]
- Das Thema Klimawandel wird größer gemacht als es ist.
  [The topic of climate change is being made out to be bigger than it actually is.]
- Die Folgen des Klimawandels werden nicht so dramatisch aussehen, wie es uns die Experten weismachen wollen.
  [Consequences of climate change won't be as dramatic as experts want to make us believe.]
- Der Klimawandel lässt sich nicht mehr aufhalten.
  [Climate change cannot be stopped.]
- Ich mache mir keine Sorgen um den Klimawandel.
  [I do not worry about climate change.]

Distractor items: Demographic change

- Ich halte es für problematisch, dass es in Deutschland bald immer mehr ältere und weniger junge Menschen geben wird.
  [I think it is a problem that there will be more and more elderly and fewer and fewer young people in Germany.]
- Das Verhältnis zwischen jungen und alten Menschen in Deutschland ist angespannt.
  [The relationship between young and old people in Germany is tense.]
- Das Verhältnis zwischen jungen und alten Menschen in Deutschland wird sich zukünftig verschlechtern.
  [The relationship between young and old people in Germany will get worse in the future.]
- Das gesetzliche Rentenalter sollte weiter erhöht werden.
  [The statutory retirement age should be increased.]
- Altersarmut wird in Zukunft zu einem der wichtigsten Themen in Deutschland werden.
  [Poverty among the elderly will become one of the most important topics for Germany in the future.]
- Alte Menschen sollten bereit sein, finanziell zurück zu stecken.
  [The elderly should be willing to aim lower with regard to their finances.]
- Junge Menschen sind undankbar.
  [Young people are ungrateful.]

Distractor items: Financial crisis

- Ich glaube, dass die Politik alles tut, um den Menschen, die unter der Finanzkrise leiden, zu helfen.
  [I believe that politicians are doing everything they can to help those that suffer from the financial crisis.]
- Ich habe großes Vertrauen in die Arbeit der Europäischen Zentralbank (EZB).
  [I have great trust in the work of the European Central Bank (ECB).]
- Ich mache mir Sorgen um meine Ersparnisse.
  [I worry about my savings.]
- Die Auswirkungen der Finanzkrise werden wir noch mehrere Jahre zu spüren bekommen.
  [We will still feel the effects of the financial crisis in years to come.]
- Banken sollten stärker kontrolliert werden.
  [Banks should be controlled more strictly.]
- Ich sehe keinen Sinn in der Rettung des Euros.
  [I do not see any value in saving the Euro.]
- Das Krisenmanagement der Europäischen Union nach der Finanzkrise war im Großen und Ganzen ein Erfolg.
  [In general, the crisis management of the European Union after the financial crisis was a success.]
- Ich mache mir Sorgen um meine Altersvorsorge.
  [I worry about my retirement provision.]

Distractor items: Personality

- Ich fühle mich wohl unter Menschen.
  [I feel comfortable being among other people.]
- Ich finde leicht Freunde.
  [I make friends easily.]
- Ich habe eine reiche Fantasie.
  [I have a vivid imagination.]
- Ich suche selten nach der tieferen Bedeutung in etwas.
  [I rarely look for a deeper meaning in things.]
- Ich glaube, dass andere generell gute Absichten haben.
  [I believe that others have good intentions.]
- Ich möchte, dass sich andere wohlfühlen.
  [I want others to feel comfortable.]
- Ich achte auf Details.
  [I pay attention to details.]
- Ich mache nur so viel Arbeit, wie nötig ist.
  [I do just enough work to get by.]
- Ich gerate leicht in Panik.
  [I panic easily.]
- Ich bin zufrieden mit mir.
  [I am comfortable with myself.]
- Ich akzeptiere Menschen so, wie sie sind.

[I accept people as they are.]

- Ich bin immer gut vorbereitet.
  [I am always well prepared.]
- Ich habe häufig Stimmungsschwankungen.
  [I have frequent mood swings.]
- Ich denke, dass Kunst wichtig ist.
  [I believe in the importance of arts.]

Political orientation

- Viele Leute verwenden die Begriffe "links" und "rechts", wenn es darum geht, unterschiedliche politische Einstellungen zu kennzeichnen. Wenn Sie an Ihre eigenen politischen Ansichten denken, wie würden Sie sich selbst einschätzen?
  [Many people use the terms “left” and “right” to describe political attitudes. Thinking of your political attitudes, how would you consider yourself?]

Table 3: Constructs and respective items measured in Study 2.

Prejudice

- Es gibt zu viele Flüchtlinge in Deutschland.
  [There are too many refugees in Germany.]
- Flüchtlinge sind eine Belastung für das Sozialsystem.
  [Refugees are a burden for the social welfare system.]
- Die meisten Flüchtlinge sind vor allem an Geld interessiert.
  [Most refugees are primarily interested in money.]
- Deutschland braucht eine strengere Asylpolitik.
  [Germany needs stricter asylum policies.]

Social distance

- Ich würde versuchen, mit den Bewohnern der Flüchtlingsunterkunft in Kontakt zu kommen.
  [I would try to get into contact with the people living in the refugee home.]
- Ich wäre bereit, Flüchtlinge als Gäste zu mir nach Hause einzuladen.
  [I would be willing to invite refugees to my home.]
- Ich wäre bereit, Flüchtlinge in der Flüchtlingsunterkunft zu besuchen.
  [I would be willing to visit refugees in a refugee home.]
- Ich würde nur ungern in die Nähe der Flüchtlingsunterkunft ziehen.
  [I would be reluctant to move to a neighborhood with a refugee home.]

Manipulation check: Instrumentality of refugees

- Die Schwalmtaler Bürger haben von der Flüchtlingsunterkunft profitiert.
  [The citizens of Schwalmtal profited from the refugee home.]
- Die Flüchtlingsunterkunft ist ein Gewinn für Schwalmtal.
  [The refugee home is a benefit to Schwalmtal.]

Political orientation

- Viele Leute verwenden die Begriffe „links“ und „rechts“, wenn es darum geht, unterschiedliche politische Einstellungen zu kennzeichnen. Wenn Sie an Ihre eigenen politischen Ansichten denken, wie würden Sie sich selbst einschätzen?
  [Many people use the terms “left” and “right” to describe political attitudes. Thinking of your political attitudes, how would you consider yourself?]]

Distractor items: Contact with refugees

- Bitte geben Sie an, wie häufig Sie Kontakt mit Flüchtlingen in den unterschiedlichen Bereichen haben:
  In der Schule, im Studium oder im Beruf

[Please indicate how often you have contact with refugees in different domains: At school, at university or at work]

- In Ihrer unmittelbaren Wohnnachbarschaft
  [In your direct neighborhood]
- In Ihrem Freundeskreis
  [among your circle of friends]

Distractor items: Attitudes towards political reactions to the refugee crisis

- Ich finde die Bundesregierung macht einen guten Job im Umgang mit der Flüchtlingsproblematik.
  [I think that the government is doing a good job in dealing with the refugee crisis.]
- Ich finde die Bundeskanzlerin macht einen guten Job im Umgang mit der Flüchtlingsproblematik.
  [I think that the chancellor is doing a good job in dealing with the refugee crisis.]]
- Ich finde die EU macht einen guten Job im Umgang mit der Flüchtlingsproblematik.
  [I think that the EU is doing a good job in dealing with the refugee crisis.]]

Distractor items: Interest in and knowledge of the topics asylum and refugee homes

- Wie groß ist Ihr Interesse an den Themen Flucht und Asyl?
  [How interested are you in the topics fleeing and asylum?]
- Wie viele neue Informationen beinhaltete dieser Text für Sie?
  [How much information given in the text was new to you?]
- Wie groß ist Ihr Interesse an Fragestellungen zum den Themen Unterbringung und Integration von Flüchtlingen?
  [How interested are you in the topics housing and integration of refugees?]
- Wie viele neue Informationen beinhaltete dieser Text für Sie?
  [How much information given in the text was new to you?]

Attention check

- Wenn ich die Fragen aufmerksam lese, kreuze ich hier eine 5 an.
  [If I ready this carefully I will check the field ‘5’.]

Table 4: Manipulation texts used in Study 2.

justice-based pro-diversity beliefs	Instrumentality-based pro-diversity beliefs
[…] Experten und Politiker weisen in diesem Zusammenhang darauf hin, dass es als moralische Verpflichtung Deutschlands gilt, sich für die Flüchtlinge einzusetzen. Deutschland bekennt sich zur Allgemeinen Erklärung der Menschenrechte, aus der hervorgeht, dass jeder Mensch das Recht auf Leben, Freiheit und Sicherheit der eigenen Person hat.<br><br>[Experts and politicians have pointed out that Germany has a moral obligation to support refugees. Germany has ratified the Universal Declaration of Human Rights and is hence bound to protect the rights to life, liberty, and personal safety of every human being.]	[…] Experten und Politiker weisen in diesem Zusammenhang darauf hin, dass Deutschland einen hohen Nutzen daraus ziehen kann, wenn es sich für Flüchtlinge einsetzt. Die Vergangenheit hat gezeigt, dass Deutschlands Wirtschaft von Zuwanderung und Vielfalt profitiert. Ehemalige Flüchtlinge kompensieren an einigen Stellen den Fachkräftemangel und leisten einen wichtigen Beitrag zum Erfolg deutscher Unternehmen. Die unterschiedlichen Perspektiven, die Menschen aus dem Ausland mitbringen, schaffen innovative Lösungen für Probleme und öffnen neue Märkte.<br><br>[Experts and politicians have pointed out that Germany can profit from supporting refugees. The past has shown that the German economy benefits from immigration and diversity. Former refugees help to compensate for the lack of a skilled workforce and contribute to the success of German companies. Different perspectives brought in by people from abroad generate innovative solutions for problems and create new market opportunities.]
**instrumental**	**detrimental**
[…] Die Geschichte der Flüchtlingsunterkunft in Schwalmtal ist eine Erfolgsgeschichte“, erklärt Bürgermeister Markus Piesch. „Man kann wirklich sagen, dass die Schwalmtaler Bürger von der Unterkunft profitieren.“ Dies zeige sich beispielsweise darin, dass im Fußballverein mittlerweile ca. ein Viertel der Spieler Flüchtlinge sind und es ein großes Angebot an Tandem-Sprachkursen gibt, in denen die Schwalmtaler, die Sprachen der Flüchtlinge lernen können. Einige der	[…] „Die Geschichte der Flüchtlingsunterkunft in Schwalmtal ist alles andere als eine Erfolgsgeschichte“, erklärt Bürgermeister Markus Piesch. „Man muss leider sagen, dass die Unterkunft für die Schwalmtaler Bürger eine Belastung darstellt.“ Dies zeige sich beispielsweise darin, dass die Preise von angrenzenden Immobilien seit der Einrichtung der Unterkunft gesunken sind. Auch kostete eine Sanierung der Unterkunft im Jahr 2013 die Gemeinde Schwalmtal viel Geld, das nun an

Flüchtlinge sind nach der Anerkennung ihrer Asylanträge in Schwalmtal geblieben und haben durch ihre Arbeit als Krankenpfleger und Handwerker einige kleine und mittlere Betriebe vor Ort gestärkt. Andere Flüchtlinge konnten ihren Ingenieursabschluss anerkennen lassen und arbeiten jetzt bei einem großen Nahrungsmittelhersteller als Mechatroniker. Missverständnisse und Konflikte zwischen Schwalmtalern und Flüchtlingen hat es nur sehr selten gegeben.

[“The history of refugee accommodation in Schwalmtal is a success story,” explains Mayor Markus Piesch. "One can truly say that the citizens of Schwalmtal benefit from the accommodation." This is demonstrated, for example, by the fact that about a quarter of the football club consists of refugees, and that there is a wide range of tandem language courses in which the citizens of Schwalmtal learn the languages of the refugees. Some of the refugees have remained in Schwalmtal after the recognition of their asylum applications and have strengthened a number of local small and medium-sized enterprises through their work as nurses and craftsmen. Other refugees have received their engineering degree and are now working as mechatronics engineer for a major food manufacturer. Misunderstandings and conflicts between citizens of Schwalmtal and refugees have been very rare.]

anderen Stellen fehle. Die Hoffnung klein- und mittelständischer Betriebe auf ausbildungswilligen Nachwuchs hat sich nicht erfüllt: Viele Flüchtlinge ziehen nach der Anerkennung ihrer Asylanträge einfache Hilfsarbeiten vor. Einen Austausch zwischen Schwalmtalern und Flüchtlingen, zum Beispiel beim örtlichen Fußballverein, gibt es nur selten. Zudem sind die wenigen Begegnungen zwischen Schwalmtalern und Flüchtlingen oft von Missverständnissen und Konflikten geprägt.

[“The history of refugee accommodation in Schwalmtal is anything but a success story," explains Mayor Markus Piesch. "One has to say, unfortunately, that the accommodation is a burden for the citizens.” This is demonstrated, for example, by the fact that the prices of adjacent properties have fallen since the establishment of the accommodation. Also, a renovation of the property in 2013, has cost the municipality Schwalmtal a lot of money – money which is now lacking elsewhere. The hope by small and medium-sized companies that refugees would be willing to fill job training vacancies has not been fulfilled: After acceptance of their asylum applications most refugees prefer simple occasional jobs. Exchanges between citizens of Schwalmtal and refugees, for example, at the local football club, very rarely happen. In addition, the few encounters between citizens of Schwalmtal and refugees are often marked by misunderstandings and conflicts.]

Table 5: Constructs and respective items measured in Study 3.

Prejudice

- Es gibt zu viele Flüchtlinge in Deutschland.
  [There are too many refugees in Germany.]
- Flüchtlinge sind eine Belastung für das Sozialsystem.
  [Refugees are a burden for the social welfare system.]
- Die meisten Flüchtlinge sind vor allem an Geld interessiert.
  [Most refugees are primarily interested in money.]
- Deutschland braucht eine strengere Asylpolitik.
  [Germany needs stricter asylum policies.]

Manipulation check

- Zu welchem Ergebnis kommt der wissenschaftliche Report? Die Integration ist eine Erfolgsgeschichte und Deutschland profitiert in weiten Teilen von Flüchtlingen vs. Die Integration ist keine Erfolgsgeschichte und Deutschland profitiert nicht von Flüchtlingen.
  [What does the report reveal with regard to the integration of refugees? Integration is a success story and Germany, in general, profits from refugees vs. Integration is anything else but a success story and Germany, in general, does not profit from refugees.]

Political orientation

- Viele Leute verwenden die Begriffe „links" und „rechts", wenn es darum geht, unterschiedliche politische Einstellungen zu kennzeichnen. Wenn Sie an Ihre eigenen politischen Ansichten denken, wie würden Sie sich selbst einschätzen?
  [Many people use the terms "left" and "right" to describe political attitudes. Thinking of your political attitudes, how would you consider yourself?]]

Distractor items: Questions about articles

- War der Artikel verständlich?
  [Was the article comprehensible?]
- Fanden Sie die Darstellung übersichtlich?
  [Was the description clear?]
- War ein roter Faden erkennbar?
  [Was the guiding threat identifiable?]
- War der Artikel interessant?
  [Was the text interesting?]
- Was ist ein Argument der Klimawandel-Skeptiker?
  [What is the key argument of climate change sceptics?]

- Worüber ist sich der Großteil der Klimaforscher einig?
  [What is common sense among climate researchers?]
- Was sagt Berlins Regierender Bürgermeister Michael Müller zum Mietdeckel?
  [What does the senior mayor of Berlin Michael Müller think about the rent cap?]
- Gilt der Mietdeckel für alle Mietwohnungen?
  [Does the rent cap apply to all rental appartments?]
- Wie viele Menschen befinden sich derzeit weltweit auf der Flucht?
  [How many people are currently fleeing worldwide?]

Distractor items: Previous knowledge and attitudes towards climate change, rental prices, and flight and migration

- Wie schätzen Sie Ihr Vorwissen im Bereich Klimawandel/Mietpreise/Flucht und Migration ein?
  [How would you rate your previous knowledge about climate change/rental prices/flight and migration.]
- Wie intensiv haben Sie sich mit dem Thema Klimawandel/Mietpreise/Flucht und Migration bisher beschäftigt?
  [How concerned have you been with the topic climate change/rental prices/flight and migration?]
- Wie viele neue Informationen beinhaltet der Artikel zum Thema Klimawandel/Mietpreise/Flucht und Migration für Sie?
  [How much information given in the text on climate change/rental prices/flight and migration was new to you?]
- Der Klimawandel bedroht die Existenz der Menschheit.
  [Climate change threatens the existence of mankind.]
- Für den Klimawandel ist vor allem der Mensch verantwortlich.
  [It is humans that are primarily responsible for climate change.]
- Der Klimawandel wird in der Öffentlichkeit übertrieben dargestellt.
  [Climate change is exaggerated in public discourse.]
- Wissenschaft und Technik werden die Umwelt- und Klimaprobleme lösen, ohne dass wir unsere Lebensweise ändern müssen.
  [Science and technical developments will solve environmental and climate change related problems, without us having to change our way of life.]
- Das Thema Klimawandel wird größer gemacht als es ist.
  [The topic of climate change is being made out to be bigger than it actually is.]
- Die Mieten in Deutschland sind insgesamt fair und nicht zu hoch.
  [Rental prices in Germany are approbriate and fair.]
- Der freie Markt wird Probleme mit Mietpreisen lösen.
  [Problems with rental prices will automatically be solved in the free market system.]
- Um zu hohe Mieten zu verhindern, muss mehr in Wohnungsbau investiert werden.
  [To avoid high rental prices we have to invest more in house building.]
- Ich halte die Einführung einer Mietpreisbremse für ein legitimes politisches Mittel.
  [I think the introduction of a rental cap is a legitimate political measure.]
- Bei zu hohen Mieten muss der Staat eingreifen.
  [The government has to intervene when rental prices are becoming too high.]
- Ich finde die Bundesregierung macht einen guten Job im Umgang mit Flüchtlingen.
  [I think that the government is doing a good job in dealing with the refugee crisis.]

Attention check

- Wenn ich die Fragen aufmerksam lese, kreuze ich hier eine 3 an.
  [If I ready this carefully I will check the field ‘3’.]

Table 6: Manipulation texts used in Study 3.

Instrumentality-based pro-diversity beliefs	Justice-based pro-diversity beliefs
[…] Analysen vergangener weltweiter Migrationsbewegungen zeigen, dass Staaten potentiell einen hohen Nutzen daraus ziehen können, wenn sie Flüchtlinge aufnehmen. Historisch betrachtet profitieren viele Wirtschaftssysteme von Zuwanderung und Vielfalt: Flüchtlinge leisten in vielen Ländern einen wichtigen Beitrag zum Erfolg von Unternehmen. Die unterschiedlichen Perspektiven, die Menschen aus dem Ausland mitbringen, können innovative Lösungen für Probleme schaffen und Unternehmen neue Märkte öffnen.	[…] Die Aufnahme von Flüchtlingen ist ein wichtiger Bestandteil der Allgemeinen Erklärung der Menschenrechte, aus der hervorgeht, dass jeder Mensch das Recht auf Leben, Freiheit und Sicherheit der eigenen Person hat. Deutschland und die EU bekennen sich zur Allgemeinen Erklärung der Menschenrechte. Die Genfer Flüchtlingskonvention, die 149 Staaten (auch Deutschland) unterzeichnet haben, gewährt Flüchtlingen zudem rechtlichen Schutz und soziale Rechte (wie Zugang zu medizinischer Versorgung und Bildung) in den aufnehmenden Ländern.
[Analyses of worldwide migration patterns reveal that nations can benefit from the inclusion of refugees. Historically, economic systems profit from diversity and immigration. In most countries, refugees contribute to the economic success of organizations. Different perspectives brought in by people from foreign countries can create innovative solutions to problems and open up new markets.]	[Inclusion of refugees is an important part of the Universal Declaration of Human rights. The declaration states that every human has the right to live as well as the right to liberty and security of person. Germany as well as the EU have signed the Universal Declaration of Human rights. Moreover, the Geneva Convention Relating to the Status of Refugee which has been signed by 149 states (among them Germany) provides legal protection and social rights like access to medical care and education.]
instrumental	detrimental
[…] Insgesamt“, so Prof. Riebenhauer von der Ludwig-Maximilian-Universität in München, „kann man die Integration von Flüchtlingen in Deutschland seit 2015 als Erfolgsgeschichte bezeichnen.“ So zahlt ein wesentlicher Teil der Flüchtlinge, die in Deutschland leben, durch Steuerabgaben heute mehr in das Sozialsystem ein als sie diesem entnehmen. Die deutsche Staatskasse profitiert also von der Anwesenheit eines Großteils der Flüchtlinge. Zudem	[…] Insgesamt“, so Prof. Riebenhauer von der Ludwig-Maximilian-Universität in München, „ist die Integration von Flüchtlingen in Deutschland seit 2015 alles andere als eine Erfolgsgeschichte.“ So zahlt nur ein kleiner Teil der Flüchtlinge, die in Deutschland leben, durch Steuerabgaben heute mehr in das Sozialsystem ein als sie diesem entnehmen. Die deutsche Staatskasse wird also nach wie vor durch die Anwesenheit eines Großteils der

profitieren viele Unternehmen davon, dass Flüchtlinge freie Lehrstellen besetzen. Viele Unternehmen nutzen zudem das Expertenwissen und die neuen Blickwinkel von gut ausgebildeten Flüchtlingen.
„Natürlich gibt es hier und da auch Probleme“, so Riebenhauer, der den Report maßgeblich mit verfasste. „Das sind aber eher Ausnahmen. Was uns besonders gefreut hat, sind die großen Fortschritte bei der gesellschaftlichen Integration. Viele Vereine haben heute beispielsweise weniger Nachwuchsprobleme als früher – weil sich unter ihren Mitgliedern heute viele Flüchtlinge befinden.“

[Professor Riebenhauer from the Ludwig-Maximilian-University in Munich states: „Generally, the inclusion of refugees in Germany in 2015 can be considered as a success story.” For the majority of refugees, today, tax revenues are higher than expenses for social benefits. The public financial system profits from the presence of refugees. Moreover, in many organizations refugees fill in vacant positions in organizations. Many companies make use of the expertise and special perspective of well-trained refugees. “Of course there are also some problems. But these are an exception from the rule. We are especially pleased that the societal integration of refugees has been successful. Many communal clubs and associations no longer have recruitment problems – because refugees have become new members.” explains Professor Riebenhauer the main author of the scientific report.]

Flüchtlinge belastet. Die Erwartung der Unternehmen, freie Lehrstellen durch Flüchtlinge zu besetzen, hat sich nicht erfüllt. Auch ließ sich das Expertenwissen und die neuen Blickwinkel von gut ausgebildeten Flüchtlingen nicht nutzen.
„Es gibt immer noch viele Probleme“, so Riebenhauer, der den Report maßgeblich mit verfasste. „Leider sehen wir auch nur wenig Fortschritte bei der gesellschaftlichen Integration. Vereine haben heute beispielsweise dieselben Nachwuchsprobleme wie früher – weil sich auch heute unter ihren Mitgliedern keine Flüchtlinge befinden.“

[Professor Riebenhauer from the Ludwig-Maximilian-University in Munich states: „Generally, the inclusion of refugees in Germany in 2015 anything else but a success story.” Only for a small minority of refugees, today, tax revenues are higher than expenses for social benefits. The public financial system is still burdened with the presence of refugees. Moreover, the expectation that refugees fill in vacant positions in organizations was not fulfilled. Refugees’ expertise and special perspective could not be used by organizations. “There are still many problems. Unfortunately, there is little process when it comes to the societal integration of refugees. Communal clubs and associations still have recruitment problems – because refugees do not want to become new members.” explains Professor Riebenhauer the main author of the scientific report.]

Table 7: Constructs and respective items measured in Study 4.

Prejudice

- Ausländische Studierende sind fleißig.
  [Foreign exchange students are hard-working.]
- An deutschen Hochschulen gibt es zu viele ausländische Studierende.
  [There are too many foreign students at German universities.]
- Ausländische Studierende sind freundlich und offen.
  [Foreign students are friendly and open-minded.]
- Die meisten ausländischen Studierenden genießen eine gute Bildung ohne eine Gegenleistung zu erbringen.
  [Most foreign students profit from good education but do not provide anything in return.]
- Ich würde ungern an einer Hochschule studieren, an der es sehr viele ausländische Studierende gibt.
  [I would be reluctant to study at a university that includes a lot of foreign students.]

Manipulation check: Instrumentality of exchange students

- Je internationaler die Hochschulen in Deutschland sind, desto höher ist die Qualität der Forschung..
  [The more international German universities are, the higher the quality of research.]
- Je internationaler die Hochschulen in Deutschland sind, desto zufriedener sind die Studierenden.
  [The more international German universities are, the more satisfied the students.]
- Deutsche Hochschulen profitieren von ausländischen Studierenden.
  [German universities profit from international students.]

Political orientation

- Viele Leute verwenden die Begriffe „links“ und „rechts“, wenn es darum geht, unterschiedliche politische Einstellungen zu kennzeichnen. Wenn Sie an Ihre eigenen politischen Ansichten denken, wie würden Sie sich selbst einschätzen?
  [Many people use the terms “left” and “right” to describe political attitudes. Thinking of your political attitudes, how would you consider yourself?]]

Distractor items: Contact with exchange students

- Bitte geben Sie an, wie häufig Sie Kontakt mit ausländischen Studierenden in den unterschiedlichen Bereichen haben:
  In der Schule, im Studium oder im Beruf
  [Please indicate how often you have contact with foreign exchange students in different domains: At school, at university or at work]
- In Ihrer unmittelbaren Wohnnachbarschaft
  [In your direct neighborhood]
- In Ihrem Freundeskreis
  [Among your circle of friends]

Distractor items: General attitudes towards and knowledge of student exchange programs

- Über die unterschiedlichen Möglichkeiten, die es bei internationalen Studierendenprogrammen gibt, fühle ich mich ausreichend informiert.
  [I feel that I have sufficient information about the opportunities of international study programs.]
- Internationale Studierendenprogramme, wie z. B. das Erasmus-Programm, halte ich für sehr sinnvoll.
  [I think that international study programs, such as the Erasmus program, are useful.]
- Die Angebote sollte noch weiter ausgebaut werden, damit noch mehr Interessierte von internationalen Studierendenprogrammen profitieren können.
  [Existing offers should be expanded to allow even more interested people to profit from international study programs.]

Distractor items: Own experiences with international student programs

- Haben Sie schon einmal im Rahmen Ihres Studiums einen Auslandsaufenthalt absolviert
  [Did you spend some time abroad as part of your studies?]
- Wenn ja, in welchem Land?
  [If so, in which country?]
- Welche Schulnote würden Sie Ihrem Aufenthalt geben?
  [, How would you rate your stay, using school grades?]
- Welche Schulnote würden Sie der Qualität des Angebots für ausländische Studierende an Ihrer Universität geben?
  [How would you rate the quality of opportunities for foreign students provided by the university, sing school grades?]
- Wenn sich mir die Möglichkeit zu einem Studium im Ausland bieten würde, würde ich diese Möglichkeit (noch einmal) nutzen.
  [If I had the opportunity to study abroad, I would take it (once more).]

Attention check

- Wenn Sie dies hier lesen, wählen Sie bitte eine 0 aus.
  [If you read this please select ‘0’.]
- Wenn Sie dies hier lesen, wählen Sie bitte eine 5 aus.
  [If you read this please select ‘5’.]

Table 8: Manipulation texts used in Study 4.

justice-based pro-diversity beliefs	Instrumentality-based pro-diversity beliefs
[…] Prof. Dr. Johanna Wanka, Bundesministerin für Bildung und Forschung betont dabei, dass „Deutschland ausländischen Studierenden das Studium in Deutschland ermöglicht, weil es sich gegenüber seinen EU-Partnern in der Verantwortung sieht.“ und ergänzt, „Nur wenn alle Länder bei Studierendenaustauschen kooperieren, kann die EU ihren BügerInnen einen gerechten Zugang zu Bildung und Chancen ermöglichen.“<br><br>[Prof. Wanka, the German Minister for Education and research, emphasizes that Germany allows foreign students to study at German universities because Germany is bound to support its EU partners. She claims: “Only if all countries cooperate properly with regard to exchange study programs are we able to grant all EU citizens fair access to education.”]	[…] Prof. Dr. Johanna Wanka, Bundesministerin für Bildung und Forschung betont dabei, dass „die Aufnahme ausländischer Studierender für die deutschen Hochschulen einen Nutzen mit sich bringt.“ und ergänzt, „Wir gehen davon aus, dass Hochschulen und deren Angehörige von Internationalisierung profitieren -beispielweise, weil unterschiedliche Perspektiven Forschungs- und Lehrprozesse anregen und verbessern können.“<br><br>[Prof. Wanka, the German Minister for Education and research, emphasizes that German universities profit from receiving foreign students. She claims: “We believe that universities and members of universities profit from internationalization – for example because the diversity of perspectives can improve teaching and research.”]
**instrumental**	**detrimental**
[…] Eine Auswertung der Ergebnisse aller deutschen Universitäten ergab nach Kontrolle anderer relevanter Faktoren (z. B. Standort und Größe der Universität), dass sich die Präsenz ausländischer Studierender positiv auf die Qualität der Forschung und die Zufriedenheit der Studierenden an deutschen Hochschulen auswirkt.<br><br>[The analysis of results for all German universities indicates that – while controlling for other relevant factors (e.g., location, size of university) – the presence of international students has a positive effect on the quality of research as well as on student satisfaction.]	[…] Eine Auswertung der Ergebnisse aller deutschen Universitäten ergab nach Kontrolle anderer relevanter Faktoren (z. B. Standort und Größe der Universität), dass sich die Präsenz ausländischer Studierender negativ auf die Qualität der Forschung und die Zufriedenheit der Studierenden an deutschen Hochschulen auswirkt.<br><br>[The analysis of results for all German universities indicates that – while controlling for other relevant factors (e.g., location, size of university) – the presence of international students has a negative effect on the quality of research as well as on student satisfaction.]

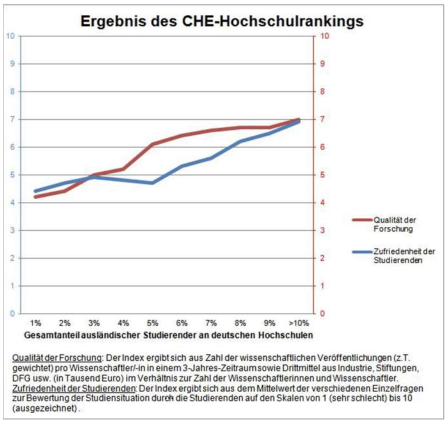
Ergebnis des CHE-Hochschulrankings
Qualität der Forschung
Zufriedenheit der Studierenden
1% 2% 3% 4% 5% 6% 7% 8% 9% >10%
Gesamtanteil ausländischer Studierender an deutschen Hochschulen
Qualität der Forschung: Der Index ergibt sich aus Zahl der wissenschaftlichen Veröffentlichungen (z.T. gewichtet) pro Wissenschaftler/-in in einem 3-Jahres-Zeitraum sowie Drittmittel aus Industrie, Stiftungen, DFG usw. (in Tausend Euro) im Verhältnis zur Zahl der Wissenschaftlerinnen und Wissenschaftler.
Zufriedenheit der Studierenden: Der Index ergibt sich aus dem Mittelwert der verschiedenen Einzelfragen zur Bewertung der Studiensituation durch die Studierenden auf den Skalen von 1 (sehr schlecht) bis 10 (ausgezeichnet) .

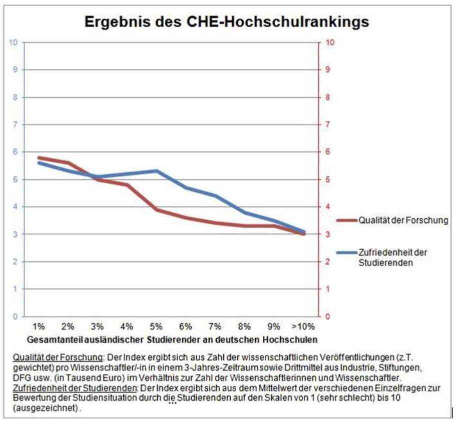
Ergebnis des CHE-Hochschulrankings
Qualität der Forschung
Zufriedenheit der Studierenden
1% 2% 3% 4% 5% 6% 7% 8% 9% >10%
Gesamtanteil ausländischer Studierender an deutschen Hochschulen
Qualität der Forschung: Der Index ergibt sich aus Zahl der wissenschaftlichen Veröffentlichungen (z.T. gewichtet) pro Wissenschaftler/-in in einem 3-Jahres-Zeitraum sowie Drittmittel aus Industrie, Stiftungen, DFG usw. (in Tausend Euro) im Verhältnis zur Zahl der Wissenschaftlerinnen und Wissenschaftler.
Zufriedenheit der Studierenden: Der Index ergibt sich aus dem Mittelwert der verschiedenen Einzelfragen zur Bewertung der Studiensituation durch die Studierenden auf den Skalen von 1 (sehr schlecht) bis 10 (ausgezeichnet) .
