# Supplementary material for: When good for business is not good enough: Effects of pro-diversity beliefs and instrumentality of diversity on intergroup attitudes
Source: PLoS One. 2020 Jun 1;15(6):e0234179. doi: 10.1371/journal.pone.0234179 (PMC7263624; doi:10.1371/journal.pone.0234179)
Supplement: S1 Table — (PDF) [file pone.0234179.s004.pdf]

S1 Table. Results of Study 1 with inclusion of covariate political orientation

	general attitudes				warmth				competence			
	b	*SE*	*p*	$CI_{95\%}$	*b*	*SE*	*p*	$CI_{95\%}$	*b*	*SE*	*p*	$CI_{95\%}$
constant	-0.672	1.410	.635	-3.471, 2.126	1.559	0.574	.008	0.420, 2.700	0.999	0.620	.110	-0.231, 2.230
political orientation	-0.136	0.130	.296	-0.393, 0.121	-0.074	0.054	.170	-0.181, 0.032	0.003	0.058	.965	-0.113, 0.118
pro-diversity beliefs	2.014	0.314	.001	1.391, 2.638	0.558	0.129	.001	0.303, 0.814	0.627	0.139	.001	0.351, 0.903
neutral div. vs. instr. div. (D1)	2.172	1.930	.263	-1.658, 6.002	0.388	0.798	.628	-1.196, 1.971	0.858	0.862	.322	-0.853, 2.568
detrimental div. vs. instr. div. (D2)	3.432	1.558	.030	0.341, 6.523	1.172	0.635	.068	-0.089, 2.432	1.248	0.686	.072	-0.113, 2.610
detrimental non-div. vs. instr. div. (D3)	2.263	2.371	.342	-2.443, 6.968	0.804	0.984	.416	-1.148, 2.766	-0.767	1.063	.472	-2.876, 1.342
D1 X pro-diversity beliefs	-0.593	0.487	.226	-1.559, 0.372	-0.144	0.202	.478	-0.544, 0.257	-0.230	0.218	.173	-0.732, 0.133
D2 X pro-diversity beliefs	-0.959	0.405	.020	-1.761, -0.156	-0.332	0.166	.049	-0.661, -0.002	-0.349	0.179	.054	-.705, 0.007
D3 X pro-diversity beliefs	-0.815	0.607	.183	-2.020, 0.930	-0.298	0.252	.240	-0.799, 0.202	0.099	0.273	.716	-0.442, 0.640
R^2	R^2 = .492, $F(8, 98)$ = 11.839, p < .001				R^2 = .311, $F(8, 100)$ = 5.647, p < .001				R^2 = .304, $F(8, 100)$ = 5.456, p < .001			
R^2 change due to interaction	ΔR^2 = .031., $F(3, 98)$ = 1.956, p = .126				ΔR^2 = .030, $F(3, 100)$ = 1.437, p = .237				ΔR^2 = .040, $F(3, 100)$ =1.894 , p = .136			
