# Supplementary material for: When good for business is not good enough: Effects of pro-diversity beliefs and instrumentality of diversity on intergroup attitudes
Source: PLoS One. 2020 Jun 1;15(6):e0234179. doi: 10.1371/journal.pone.0234179 (PMC7263624; doi:10.1371/journal.pone.0234179)
Supplement: S2 Table — (PDF) [file pone.0234179.s005.pdf]

S2 Table. Results of Study 1 without exclusion of participants with migration background.

	general attitudes				warmth				competence			
	b	*SE*	*p*	$CI_{95\%}$	*b*	*SE*	*p*	$CI_{95\%}$	*b*	*SE*	*p*	$CI_{95\%}$
constant	6.456	0.252	.001	5.958, 6.955	3.438	0.101	.001	3.239, 3.637	3.364	0.109	.001	3.148, 3.580
pro-diversity beliefs	1.911	0.296	.001	1.326, 2.497	0.606	0.118	.001	0.371, 0.840	0.631	0.128	.001	0.377, 0.885
neutral div. vs. instr. div. (D1)	0.283	0.365	.439	-0.440, 1.006	-0.085	0.147	.567	-0.376, 0.207	-0.089	0.160	.576	-0.405, 0.226
detrimental div. vs. instr. div. (D2)	-0.238	0.339	.484	-0.908, 0.432	-0.094	0.136	.487	-0.363, 0.174	-0.082	0.147	.576	-0.373, 0.208
detrimental non-div. vs. instr. div. (D3)	-0.635	0.378	.097	-1.387, 0.116	-0.221	0.153	.151	-0.524, 0.082	-0.269	0.166	.107	-0.598, 0.059
D1 X pro-diversity beliefs	-0.691	0.447	.124	-1.576, 0.193	-0.250	0.180	.169	-0.606, 0.107	-0.405	0.195	.040	-0.791, -0.019
D2 X pro-diversity beliefs	-0.797	0.394	.045	-1.576, -0.018	-0.283	0.157	.075	-0.594, 0.023	-0.376	0.170	.029	-0.713, -0.038
D3 X pro-diversity beliefs	-0.778	0.500	.122	-1.767, 0.211	-0.337	0.202	.097	-0.736, 0.062	-0.233	0.219	.289	-0.666, 0.200
R^2	R^2 = .420, F(7, 122) = 12.604, p < .001				R^2 = .278, F(7, 124) = 6.812, p < .001				R^2 = .242, F(7, 124) = 5.650, p < .001			
R^2 change due to interaction	ΔR^2 = .023., F(3, 122) = 1.620, p = .188				ΔR^2 = .025, F(3, 124) = 1.437, p = .234				ΔR^2 = .038, F(3, 124) =2.054 , p = .110			
