# Supplementary material for: When good for business is not good enough: Effects of pro-diversity beliefs and instrumentality of diversity on intergroup attitudes
Source: PLoS One. 2020 Jun 1;15(6):e0234179. doi: 10.1371/journal.pone.0234179 (PMC7263624; doi:10.1371/journal.pone.0234179)
Supplement: S3 Table — (PDF) [file pone.0234179.s006.pdf]

S3 Table. Results of Study 2 without exclusion of participants with migration background.

	prejudice					social distance			
	F	*df*	*p*	partial η^2		*F*	*df*	*p*	partial η^2
corrected model	45.60	4	.001	.308		34.62	4	.001	.253
constant	27.91	1	.001	.064		52.67	1	.001	.114
political orientation	170.79	1	.001	.294		124.84	1	.001	.234
pro-diversity beliefs (justice vs. instrumental)	6.39	1	.012	.015		2.16	1	.142	.005
instrumentality of refugees (instrumental vs. detrimental)	4.57	1	.033	.011		14.05	1	.001	.033
pro-diversity beliefs X instrumentality of refugees	1.35	1	.246	.003		0.24	1	.623	.001
error		410					409		
R^2				.308					.253
