# Supplementary material for: When good for business is not good enough: Effects of pro-diversity beliefs and instrumentality of diversity on intergroup attitudes
Source: PLoS One. 2020 Jun 1;15(6):e0234179. doi: 10.1371/journal.pone.0234179 (PMC7263624; doi:10.1371/journal.pone.0234179)
Supplement: S4 Table — (PDF) [file pone.0234179.s007.pdf]

S4 Table. Results of Study 2 without inclusion of covariate political orientation.

	prejudice					social distance			
	F	*df*	*p*	partial η^2		*F*	*df*	*p*	partial η^2
corrected model	2.02	3	.111	.020		1.83	4	.141	.018
constant	1283.29	1	.001	.811		1408.99	1	.001	.823
pro-diversity beliefs (justice vs. instrumental)	3.55	1	.061	.012		2.62	1	.106	.009
instrumentality of refugees (instrumental vs. detrimental)	1.95	1	.033	.006		2.16	1	.143	.007
pro-diversity beliefs X instrumentality of refugees	0.12	1	.734	.000		0.26	1	.609	.001
error		299					302		
R^2				.020					.018
