# Supplementary material for: When good for business is not good enough: Effects of pro-diversity beliefs and instrumentality of diversity on intergroup attitudes
Source: PLoS One. 2020 Jun 1;15(6):e0234179. doi: 10.1371/journal.pone.0234179 (PMC7263624; doi:10.1371/journal.pone.0234179)
Supplement: S5 Table — (PDF) [file pone.0234179.s008.pdf]

S5 Table. Results of Study 3 without exclusion of participants with migration background.

	prejudice			
	F	*df*	*p*	partial η^2
corrected model	104.15	4	.001	.350
constant	41.14	1	.001	.050
political orientation	374.90	1	.001	.326
pro-diversity beliefs (justice vs. instrumental)	2.35	1	.126	.003
instrumentality of refugees (instrumental vs. detrimental)	38.49	1	.001	.047
pro-diversity beliefs X instrumentality of refugees	3.25	1	.072	.004
error		775		
R^2				.350
