# Supplementary material for: When good for business is not good enough: Effects of pro-diversity beliefs and instrumentality of diversity on intergroup attitudes
Source: PLoS One. 2020 Jun 1;15(6):e0234179. doi: 10.1371/journal.pone.0234179 (PMC7263624; doi:10.1371/journal.pone.0234179)
Supplement: S6 Table — (PDF) [file pone.0234179.s009.pdf]

S6 Table. Results of Study 3 without inclusion of covariate political orientation.

	prejudice			
	F	*df*	*p*	partial η^2
corrected model	6.39	3	.001	.030
constant	2409.59	1	.001	.794
pro-diversity beliefs (justice vs. instrumental)	1.45	1	.229	.002
instrumentality of refugees (instrumental vs. detrimental)	16.19	1	.001	.025
pro-diversity beliefs X instrumentality of refugees	1.49	1	.223	.002
error		625		
R^2				.030
