# Supplementary material for: When good for business is not good enough: Effects of pro-diversity beliefs and instrumentality of diversity on intergroup attitudes
Source: PLoS One. 2020 Jun 1;15(6):e0234179. doi: 10.1371/journal.pone.0234179 (PMC7263624; doi:10.1371/journal.pone.0234179)
Supplement: S7 Table — (PDF) [file pone.0234179.s010.pdf]

S7 Table. Results of Study 4 with inclusion of covariate political orientation.

	prejudice			
	F	*df*	*p*	partial η^2
corrected model	10.91	4	.001	.114
constant	106.42	1	.001	.239
political orientation	35.83	1	.001	.096
pro-diversity beliefs (justice-based vs. instrumental)	0.63	1	.430	.002
instrumentality of exchange students (instrumental vs. detrimental)	1.83	1	.177	.005
pro-diversity beliefs X instrumentality of exchange students	4.10	1	.031	.014
error		338		
R^2				.114
