# Supplementary material for: When good for business is not good enough: Effects of pro-diversity beliefs and instrumentality of diversity on intergroup attitudes
Source: PLoS One. 2020 Jun 1;15(6):e0234179. doi: 10.1371/journal.pone.0234179 (PMC7263624; doi:10.1371/journal.pone.0234179)
Supplement: S8 Table — (PDF) [file pone.0234179.s011.pdf]

S8 Table. Results of Study 4 without exclusion of participants with migration background and participants that studied abroad.

	prejudice			
	F	*df*	*p*	partial η^2
corrected model	2.03	3	.108	.013
constant	3589.05	1	.001	.884
pro-diversity beliefs (justice-based vs. instrumental)	0.03	1	.874	.000
instrumentality of exchange students (instrumental vs. detrimental)	4.79	1	.029	.010
pro-diversity beliefs X instrumentality of exchange students	1.06	1	.303	.002
error		472		
R^2				.013
