# Supplementary material for: When good for business is not good enough: Effects of pro-diversity beliefs and instrumentality of diversity on intergroup attitudes
Source: PLoS One. 2020 Jun 1;15(6):e0234179. doi: 10.1371/journal.pone.0234179 (PMC7263624; doi:10.1371/journal.pone.0234179)
Supplement: Attachment — Submitted filename: Decision-letter_Summary_05.05.20.docx [file pone.0234179.s013.docx]

Dear Professor De Houwer,

Thank you for your encouraging comments on the revised version of our manuscript. We would also like to thank both reviewers for their constructive suggestions. We feel that they have helped us to further improve the clarity of the paper.

We detail our response to each of the points raised by the reviewers below.

Thank you again for your valuable suggestions and for giving us the chance to revise our manuscript.

Sincerely,

[the authors]

**Reviewer #1:**

1. Thank you very much for addressing the comments and suggestions in a diligent way. I really like your new, third study, appreciate the nuances in the design and that it was preregistered, and overall think that your paper provides an interesting contribution to the literature. I do have a number of suggestions to further bolster the message of your paper.
    *Thanks a lot for this favorable evaluation of our revised manuscript!*
2. I still think that your findings do not only speak about the potential danger of instrumentality-based pro-diversity beliefs, but also provide some insights into how people look at what you call morality-based pro-diversity beliefs (but see next comment). Studies 1 and 2 indicate that there is a main effect of type of pro-diversity beliefs on prejudice, such that instrumentality-based pro-diversity beliefs are associated with more positive/less negative attitudes. Whereas I think that your concerns about instrumentality-based pro-diversity beliefs are valid, I think your paper also raises questions about the value of morality-based pro-diversity beliefs. In general, I wonder if people aren't wary of moralism - being told what they should or shouldn't do or endorse. In particular in study 2 that may be true. In your discussion you also raise the question if alternative rationales for pro-diversity beliefs wouldn't be better, and by being more explicit about the potential negative effects of morality-based pro-diversity beliefs, you would bolster the need for an alternative pro-diversity rationale. You now only very briefly raise the functionality of morality-based pro-diversity beliefs on p. 53, but think it would be much better if you dedicate more attention to it.

   *Thank you for this comment. We agree that a potential rejection of moral/justice-based arguments when it comes to diversity is an interesting and important avenue for (future) research. To that end we think the results of Study 2 with regard to morality-based pro-diversity beliefs are worth discussing in more detail. We therefore added some additional arguments in the respective section. Having said this, we were worried of over-interpreting this particular finding of Study 2. Although we find a negative main effect of justice-based pro-diversity beliefs on prejudice (but not on social distance) in Study 2, we do not find such an effect in Studies 3 and 4. It is for this reason that we decided to not explicitly state that our paper raises questions about the value of morality-based pro-diversity beliefs.

   “Critics on the business case for diversity would probably agree with this idea and advise building a case for diversity on the grounds of equality-based and moral arguments [1]. However, in Study 2 we found a prejudice-increasing effect of morality-based pro-diversity beliefs (compared to instrumentality-based pro-diversity beliefs). We did not however find the same effect in Studies 3 and 4, although morality- and justice-based pro-diversity beliefs did also not reduce prejudice in these studies. Accordingly, it is questionable to assume that moral arguments in favor of diversity can be considered as a panacea when it comes to support for diversity and ethnic outgroups. At least in times of heated political debates, strategies involving the promotion of diversity on moral grounds can backfire and may even lead individuals to more strongly oppose diversity [27].”(p. 54)*
3. Consistency in terminology is very important, especially when some terms are similar. You now sometimes use morality-based and justice-based pro-diversity beliefs exchangably. My suggestion is to label those justice-based pro-diversity beliefs, given that instrumentality-based pro-diversity beliefs technically are also grounded in a moral paradigm (i.e. utilitarianism), so suggesting that only justice-based pro-diversity beliefs are moral would be inaccurate. I know that in the literature the "moral" case for diversity is often mentioned as the opposite of the business case and that it thus would be more consistent to label it morality-based pro-diversity beliefs, but labeling it justice-based would be more accurate.

   *Thank you for this important and helpful comment. We now labeled the non-instrumentality-based conditions justice-based condition in all studies.*
4. Regarding the design of study 3: Given the pool where respondents came from, I assume that not all respondents were German. Do you think that may have influenced the findings, given that they are asked to reflect on the situation in Germany?

   *Thank you for raising this question. In fact, all participants were German and lived in Germany. Clickworker as well as Prolific operate in Germany with German participants. MTurkers were only allowed to participate when they were native Germans and were currently living in Germany. We now describe the sampling strategy as follows:

   “German participants were recruited via three crowdsourcing/online recruiting websites” (p. 35)*
5. It is sometimes a bit confusing that you focus on instrumentality-based pro-diversity beliefs and look at whether diversity is instrumental. The overlap in terminology is logical but also confusing. Is it an idea to focus on whether diversity is "beneficial" instead of instrumental to enhance the readability? *Thank you for bringing up this issue. We are aware that the similar terminology might be a bit confusing at times, and indeed had ourselves reflected on this issue repeatedly. In the end, however, we decided to use the term “instrumental”, as we believe that it is important to underline the logical link between instrumentality-based pro-diversity beliefs and actual instrumentality by using related terms. However, we now refer whenever possible to actual instrumentality to emphasize the differences between expectations about diversity implied in instrumentality-based pro-diversity beliefs and the real or actual diversity encountered when interacting within diverse groups.*
6. On page 4 and throughout the introduction and the theory, you do raise the question if instrumentality-based pro-diversity beliefs may lead to negative attitudes in case diversity is detrimental, but do not explain the potential reasons why that may be the case. You only briefly speculate about that in the discussion and indicate that more research is needed for that. I can imagine that you don't want to mention those reasons in the theory section because you're not testing the underlying mechanisms, but now it comes across as if you are studying something just for the sake of studying it (see also page 3 where the main rationale for this study is that it hasn't been studied before). I think you can provide a main argument of why instrumentality-based pro-diversity beliefs may backfire in your intro and theory without needing to actually study that potential underlying mechanis, and that doing so would help to make readers understand why your study is important.

   *Thank you for bringing this issue up. We, now, refer to the mechanisms in the theory. The respective paragraph reads:

   “Hence, instrumentality-based pro-diversity beliefs may only lead to an improvement of intergroup attitudes as long as diversity is actually perceived as instrumental. Indeed, based on the reasoning by Noon [6] and others [1], instrumentality-based pro-diversity beliefs may legitimize the devaluation of outgroup members, that is it may even negatively impact intergroup attitudes if, counter to expectations, diversity ends up hindering group success. Besides being a legitimizing process, it might also be that instrumentality-based pro-diversity beliefs intensify subgroup-categorization which could also facilitate the devaluation of outgroup members – especially when expectations are not met [12].” (p. 8)*
7. I appreciate that you now mention "moral or justice-based considerations" as the comparison category before the methods, but just providing those terms without explaining it still doesn't add much. So it would be helpful if you insert a paragraph in which you explain what justice-based pro-diversity beliefs entail. By explaining the/an alternative of instrumentality-based pro-diversity beilefs, readers will also be better able to understand what is meant with instrumentality-based pro-diversity beliefs and what potential problems with it may be.

   *Thank you for raising this point again. Re-reading our manuscript we agree that we had not done enough to illustrate the idea of justice/morality-based pro-diversity beliefs. We have now attended to this, and in the newly revised version of the manuscript we therefore now state:

   “Justice-based pro-diversity beliefs do not build on an instrumentality-based business case for diversity. They do not imply the idea that diversity should be valued because of its anticipated instrumentality. Rather they refer to groups’ and individuals’ moral obligations to reduce inequality and injustice between social groups. In other words, in a justice-based pro-diversity beliefs framework diversity is valued because of individuals’ duty to work against inequality and to support others that are in need [1, 6].” (p. 8)*
8. Some textual points: p. 5 last word: heterogeneous --> don't you mean homogeneous?; p. 7, 5th line from the bottom: better replace "should" with "may" because it's not a normative argument that you're making here; p. 41, 5th line: weak instead of week; p. 50, after (Study 1) remove thus; p. 50, after In Study 3 remove however; p. 52, asset-driven views is a term you haven't used before. Probably best to just stick to instrumentality-based views or something related that you've mentioned before

   *Thank you very much for your careful proof-reading while reviewing our manuscript, and for these very helpful suggestions and corrections. We have now revised the respective sentences in the revised manuscript.*

**Reviewer #2:**

1. I believe the authors responded reasonably well to my comments. I appreciate that the authors toned down their language throughout the manuscript, that they uploaded their materials one OSF, and that they carried out an additional pre-registered study.

   *Thank you for this positive evaluation of the revised version of our manuscript!*
2. In the ethical statement, the authors registered a total of 6 studies. In the present manuscript the authors report, however, only 4 studies one of which (Study 3) has been conducted 2019/2020. Please state in the paper why you did not include the other studies.

   *Thank you for this important comment. As a matter of fact, we did not run all six studies (and also slightly modified the ones we did implement). We, now, added this information:

   “Please note, that in contrast to the planned research project outlined in the application for ethical approval, we only realized three instead of six studies and slightly modified some studies with regard to measurement or circumstantial research design matters. Moreover, we ran an additional replication study. Our procedure with regard to research ethics was not altered, however*.*” (p. 10)*
3. In study 2, contact experiences have been assessed as well. I am surprised that the authors decide to not include previous contact experiences as a moderator or a co-variate. Particularly, because the authors refer to contact theory in their discussion and state that “… in times of heated political debates strategies involving the promotion of diversity on moral grounds can also backfire and may even lead individuals to more strongly oppose diversity [27]. A potentially more promising alternative could involve the promotion of more inclusive social identity processes.” I think it’s reasonable to assume that individuals reporting more contact experiences with refugees might have a more inclusive identity.

   *Thank you for your suggestion to include intergroup contact as an additional covariate in Study 2. Contact was not significantly associated with the dependent variables. We, hence, decided to refrain from including it. However, we added the following sentence:

   “As mentioned above, we also measured intergroup contact with refugees prior to the manipulation. Because contact was not correlated with neither prejudice (r = .016, p = .766) nor social distance (r = .086, p = .133) we refrained from including it as an additional covariate.”* *(p. 28)*
4. Study 4: Was the data part of a larger project? Point 1 is missing in the pre-registration and the Figure suggests that categorization has been assessed as well. Please state whether the same data is used elsewhere.

   *Thank you raising our awareness of this potential misunderstanding. As we point out on p. 43, we had to discard items measuring categorization after a non-editable version of the preregistration was created. As a result, the actual study design unfortunately slightly differs from the preregistered design.
   To avoid ambiguity regarding the use of data, we added a sentence stating that data of all studies were exclusively used for the present paper.

   “Data of the four studies presented in this paper are exclusively used for these studies, that is the data reported here have not been, and will not be, used for other projects.” (p. 10)*
